# Nebulin nemaline myopathy recapitulated in a compound heterozygous mouse model with both a missense and a nonsense mutation in *Neb*

**DOI:** 10.1186/s40478-020-0893-1

**Published:** 2020-02-17

**Authors:** Jenni M. Laitila, Elyshia L. McNamara, Catherine D. Wingate, Hayley Goullee, Jacob A. Ross, Rhonda L. Taylor, Robbert van der Pijl, Lisa M. Griffiths, Rachel Harries, Gianina Ravenscroft, Joshua S. Clayton, Caroline Sewry, Michael W. Lawlor, Coen A. C. Ottenheijm, Anthony J. Bakker, Julien Ochala, Nigel G. Laing, Carina Wallgren-Pettersson, Katarina Pelin, Kristen J. Nowak

**Affiliations:** 1 Folkhälsan Institute of Genetics, Folkhälsan Research Center, Biomedicum, Helsinki, Finland; 2 Department of Medical and Clinical Genetics, Medicum, University of Helsinki, Helsinki, Finland; 3 Harry Perkins Institute of Medical Research, QEII Medical Centre, Nedlands, Western Australia Australia; 4 Centre for Medical Research, University of Western Australia, Perth, Australia; 5 School of Human Sciences, University of Western Australia, Perth, Western Australia Australia; 6 Centre for Human and Applied Physiological Sciences / Randall Centre for Cell and Molecular Biophysics, School of Basic and Medical Biosciences, Faculty of Life Sciences and Medicine, King’s College London, London, UK; 7 Department of Cellular and Molecular Medicine, University of Arizona, Tucson, USA; 8 Department of Neuropathology, PathWest Anatomical Pathology, Nedlands, Western Australia Australia; 9 Dubowitz Neuromuscular Centre, Institute of Child Health and Great Ormond Street Hospital, Guilford Street, London, UK; 10 Wolfson Centre of Inherited Neuromuscular Diseases, RJAH Orthopaedic Hospital, Oswestry, UK; 11 Division of Pediatric Pathology and Neuroscience Research Center, Medical College of Wisconsin, Milwaukee, USA; 12 Faculty of Biological and Environmental Sciences, Molecular and Integrative Biosciences Research Programme, University of Helsinki, Helsinki, Finland; 13 School of Biomedical Sciences, Faculty of Health and Medical Sciences, University of Western Australia, Nedlands, Australia; 14 Office of Population Health Genomics, Public and Aboriginal Health Division, Western Australian Department of Health, East Perth, Western Australia Australia

**Keywords:** Nebulin, Murine model, Nemaline myopathy, Skeletal muscle, Neuromuscular disease, Congenital myopathy

## Abstract

Nemaline myopathy (NM) caused by mutations in the gene encoding nebulin (*NEB*) accounts for at least 50% of all NM cases worldwide, representing a significant disease burden. Most *NEB*-NM patients have autosomal recessive disease due to a compound heterozygous genotype. Of the few murine models developed for *NEB*-NM, most are *Neb* knockout models rather than harbouring *Neb* mutations. Additionally, some models have a very severe phenotype that limits their application for evaluating disease progression and potential therapies. No existing murine models possess compound heterozygous *Neb* mutations that reflect the genotype and resulting phenotype present in most patients. We aimed to develop a murine model that more closely matched the underlying genetics of *NEB*-NM, which could assist elucidation of the pathogenetic mechanisms underlying the disease. Here, we have characterised a mouse strain with compound heterozygous *Neb* mutations; one missense (p.Tyr2303His), affecting a conserved actin-binding site and one nonsense mutation (p.Tyr935*), introducing a premature stop codon early in the protein. Our studies reveal that this compound heterozygous model, *Neb*^Y2303H, Y935X^, has striking skeletal muscle pathology including nemaline bodies. In vitro whole muscle and single myofibre physiology studies also demonstrate functional perturbations. However, no reduction in lifespan was noted. Therefore, *Neb*^Y2303H,Y935X^ mice recapitulate human *NEB*-NM and are a much needed addition to the *NEB*-NM mouse model collection. The moderate phenotype also makes this an appropriate model for studying *NEB*-NM pathogenesis, and could potentially be suitable for testing therapeutic applications.

## Introduction

Nemaline myopathy (NM) is one of the most common congenital myopathies and is caused by pathogenic variants in one of at least twelve different genes [4, 18, 27, 29, 37, 52, 55, 64, 69, 74, 75, 91]. Patient muscle biopsies show accumulation of Z-disc and thin filament associated proteins into aggregates called nemaline bodies, usually accompanied by disorganization of the muscle Z discs [14, 80, 83]. There can be large variation in clinical severity, from in utero presentation and early neonatal death, through to milder forms with later onset [77, 86].

Autosomal recessive NM is most commonly caused by pathogenic variants in the nebulin gene (*NEB;* NEM2, Online Mendelian Inheritance in Man #256030) [44]. While the clinical severity varies from severe, early onset forms through to milder forms, *NEB*-NM most often presents as a slowly progressive disease, with weakness in proximal skeletal muscles and potential later involvement of distal muscles [77]. Appropriate respiratory management usually results in a normal lifespan [73]. To date, over 200 NM-causing pathogenic variants have been identified throughout the entire length of *NEB* [32, 44, 64, 65]. Variants in *NEB* can also cause disorders described as distal nebulin myopathy [84], distal NM [45], foetal akinesia/lethal multiple pterygium syndrome [1, 44] and, in rare cases, core-rod myopathy [71].

*NEB* is comprised of 183 exons that encode a theoretical 26 kb full-length mRNA, although extensive alternative splicing results in a wide variety of different transcripts [19, 38]. Nebulin is a giant (600–900 kDa) actin-binding protein, with the C-terminus located deep within the Z disc whilst the rest of the protein stretches nearly the entire length of the thin filament of the skeletal muscle sarcomere [36]. It is thought to stabilise, stiffen and strengthen actin filaments, specify minimum thin-filament length as well as regulate Z-disc width and intermyofibrillar connectivity through its interaction with multiple proteins, e.g. desmin, titin and myopalladin [8, 13, 31, 34, 62, 81, 89]. Recently it has also been shown that nebulin contributes to thin filament activation and cross-bridge recruitment [34]. Although these multiple roles for nebulin have been suggested, many known and potential aspects of its function are still to be understood. For example, the dynamic movement of nebulin and its interaction partners in the thin filament during muscle contraction remains unclear [13, 88]. Furthermore, the nebulin transcript is alternatively spliced to produce alternative protein isoforms (for example super repeat S21 in isoforms a and b; see our recent report [40]). The functional importance of the different isoforms is currently under investigation.

Despite the large number of pathogenic variants identified in *NEB*, no clear mutational hotspots or genotype-phenotype correlations have been found [44], and the functional significance of disease-causing variants are largely unknown. Most *NEB*-NM patients have a compound heterozygous genotype and, if one of the two variants is missense, then the other is a more disruptive variant such as a nonsense variant or a deletion/insertion [44]. Interestingly, the same variant has been identified in patients with diverse clinical severities, or even with different myopathies [44]. As such, an appropriate model organism is required to better understand the complex pathogenetic mechanisms that underlie the diverse forms of NM. Such a model would also enable potential therapies to be tested in a system that recapitulates the human disease.

A number of murine models have been published [8, 25, 46, 47, 61, 89, 90], and have each provided new knowledge about nebulin function and the potential pathogenesis of NM. However, none of these models possess a compound heterozygous genotype that would be representative of most human *NEB*-NM cases. Furthermore, there are currently no *Neb* models that recapitulate the most common phenotype of *NEB*-NM. Therefore, we have produced a murine model with compound heterozygous *Neb* variants; a missense variant (p.Tyr2303His) in the perfectly conserved tyrosine residue in an actin-binding site, and a nonsense variant (p.Tyr935*) introducing a premature stop codon in the beginning of the super-repeat region. Our aims were to characterise this new murine model, and to investigate how accurately it recapitulates the phenotype of most patients with *NEB*-NM.

## Materials & methods

### C57BL/6J-*Neb*^Y2303H,Y935X^ mice

Mouse lines with a C57BL/6J background carrying either the *Neb*^Y2303H^ or the *Neb*^Y935X^ variant were selected from a missense mutation library derived from N-ethyl-N-nitrosourea (ENU) mutagenesis (Australian Phenomics Facility, Australian National University, Canberra [5]) on the basis of their potential pathogenicity. The missense variant NP_035019.1:p.(Tyr2303His), c.6907 T > C (NM_010889.1), changed the perfectly conserved amino acid tyrosine (Y, Tyr) in the last actin-binding site (SDxxYK) of the eighth super repeat (S8). The nonsense variant NP_035019.1:p.(Tyr935*), c.2805C > G, introduced a premature stop codon in the third super repeat (S3), which was expected to lead to nonsense-mediated RNA decay. To maintain the parental lines, mice heterozygous for each variant, either C57BL/6J-*Neb*^Y2303H(+/−)^ or C57BL/6J-*Neb*^Y935X(+/−)^, were bred with heterozygous mice of the same genotype, giving rise to homozygous, heterozygous and wild-type genotypes. Resulting mice that were heterozygous for each variant were bred together to generate compound heterozygous mice C57BL/6J-*Neb*^Y2303H(+/−),Y935X(+/−)^, hereafter annotated *Neb*^Y2303H,Y935X^. This breeding regime was chosen as homozygous mice for the missense mutation (*Neb*^Y2303H(+/+)^) were less fertile than those that were heterozygous (*Neb*^Y2303H(+/−)^), and mice homozygous for the nonsense variant (*Neb*^Y935X(+/+)^) were not viable. *Neb*^Y2303H,Y935X^ mice were compared against wild-type littermates or age- and sex-matched C57BL/6J mice.

Mice were housed in a pathogen-free facility at the Animal Resources Centre (Murdoch, Western Australia) and were cared for according to guidelines set by the National Health and Medical Research Council of Australia. Rooms were on a 15:9 h light/dark cycle and mice had ad libitum access to tap water and a regular diet (Specialty Feeds, Western Australia).

Experimentation was approved by the Animal Ethics Committees of the Animal Resources Centre and The University of Western Australia.

### In vivo phenotypic tests

The compound heterozygous *Neb*^Y2303H,Y935X^ mice, and the heterozygous *Neb*^Y2303H(+/−)^ and *Neb*^Y935X(+/−)^ mice were analysed against wild-type littermate controls of the same age and sex. Bodyweight was measured at 3 and 6 months.

#### Voluntary exercise

Mice were housed individually with access to voluntary low-profile wireless running wheels (ENV-044, Med Associates Inc., Fairfax, VT, USA) for 6 consecutive days at 3 and 6 months of age. A wireless USB Interface Hub (DIG-804, Med Associates) was used to collect wheel data that were viewed and extracted using Wheel Manager (SOF-860, Med Associates). Four parameters were calculated for daily performance, including: daily distance travelled, time spent running, average speed and maximum speed. Only data from days 4 to 6 were used for analysis to allow for initial acclimatisation to the wheel.

#### Grip strength

At 3 and 6 months of age, each mouse was lifted by its tail until its front paws were in line with the bar of the grip strength meter (Bioseb, Vitrolles, France). Mice were then allowed to reach out to the bar before being gently pulled away at a slow, constant speed. This allowed mice to build up resistance until the grasp was finally broken, at which point the grip strength value (N) was recorded. The test was repeated three times for each mouse. Measurements were discarded if the mouse used only one paw, also used its hind paws, turned backwards during the pull, or left the bar without resistance.

#### Rotarod

The day before testing (or as close as possible), mice were acclimatised to the rotarod (Ugo Basile 47,600, Schwenksville, PA, USA) by training for 2 min with slow rod rotation (4 rpm). Mice that fell off during this period were replaced onto the rod until the full time had elapsed. To test performance, mice were placed on the rotarod set at 4 rpm with speed of rotation gradually increased to a maximum of 60 rpm over a period of 3 min. The latency (time to fall) and the speed at this point were recorded. Mice that fell off within the first 10 s were re-tested after a rest of at least 10 min. However, mice did not get re-tested if they performed a passive rotation (the mouse held on and spun around the rod). When mice did not fall off the rotarod after 5 min, the experiment was ceased. The test was repeated three times within the same session, with each mouse given at least 5 min to rest between each test.

### Transcript level

*Neb* transcript expression in 9–12 month-old gastrocnemius samples from female *Neb*^Y2303H,Y935X^ mice was compared with expression in the parental lines (*Neb*^Y2303H(+/+)^, *Neb*^Y2303H(+/−)^ and *Neb*^Y935X(+/−)^) and the C57BL/6J background strain. Skeletal muscle samples from *Neb*^Y935X(+/+)^ mice were not included due to their embryonic or early neonatal death, and also as the focus of this study was characterising the compound heterozygous *Neb* mutant mice.

#### RNA extraction and cDNA synthesis

Snap frozen gastrocnemius muscles were divided longitudinally, and half (~ 20–30 mg) was homogenised in 300 μl Buffer RLT with β-mercaptoethanol using a BioSpec minibead beater (maximum oscillations per min at 30 s intervals). RNA was subsequently extracted using an RNeasy fibrous tissue mini kit with on-column DNase I treatment (QIAGEN, USA). RNA yield was determined using a Nanodrop ND-1000 spectrophotometer and quality assessed on a 1% agarose gel. Up to 1 μg of RNA was used for cDNA synthesis using the SuperScript III first-strand synthesis system with random hexamer primers (Thermo Fisher, USA). Samples were diluted tenfold for 1 μg of starting RNA and scaled accordingly for lower input quantities.

#### Quantitative RT-PCR

Transcript abundance of target genes was determined by quantitative reverse-transcriptase PCR (qRT-PCR) using the Rotor-Gene SYBR Green PCR kit (QIAGEN, USA) and a Rotor-Gene Q cycler (QIAGEN, USA). Reactions were performed in 10 μl volumes with 0.8 μM primers and 1 μl diluted cDNA.

The relative abundance of mutant (p.Tyr2303His, c.6907 T > C, C allele) and wild-type (T allele) *Neb* transcripts was determined using allele-specific qRT-PCR. Forward primers were specific for either the wild-type (5′- GGACATTGCTAGTGACTTTAAAT) or Tyr2303His mutant allele (5′- GGACATTGCTAGTGACTTTAAAC) and used in combination with a non-distinguishing reverse primer (5′- CACAGGGCTGGTGTATTTGG). The wild-type allele-specific forward primer also detected transcripts from the mutant p.Tyr935* allele. Standards for assessment of qPCR efficiency were generated by serial dilution of cDNA of the respective genotypes. Cycling conditions were as follows: 95 °C for 5 min, 45 cycles of 95 °C for 10 s and 62 °C for 15 s, followed by melt curve analysis.

Relative abundance was calculated using the ΔCt method with *Tbp* (F: 5′-ATCTACCGTGAATCTTGGCTGT, R: 5′-TGTTCTTCACTCTTGGCTCCTG) and *Eef2* (F: 5′-AGAAAGCCAACATCCGGAACA, R: 5′-GATGGCGGTGGATTTGATTGT) for normalisation. Cycling conditions were as follows: 95 °C for 5 min, 45 cycles of 95 °C for 10 s and 60 °C for 15 s, followed by melt curve analysis.

### Protein electrophoresis

Nebulin protein content was analysed using the 1% SDS agarose gel method [66]. Quadriceps muscle tissue from three female mice per genotype was ground to a fine powder using Dounce homogenisers cooled in liquid nitrogen and acclimated to − 20 °C for 30 min. Tissue powder was resuspended in a 1:1 mixture of an 8 M urea buffer (in M; 8 urea, 2 thiourea, 0.05 Tris-HCl, 0.075 dithiothreitol, as well as 3% SDS and 0.03% bromophenol blue, pH 6.8) and 50% glycerol containing protease inhibitors (in mM; 0.04 E-64, 0.16 leupeptin, and 0.2 PMSF). Tissue powder was homogenised in solution for 4 min, followed by a 10 min incubation at 60 °C. Samples were centrifuged at 12,000 rpm and the supernatant was divided into smaller aliquots and flash frozen for storage at − 80 °C. SDS-agarose (SDS-AGE) 1% gels, run in a Hoefer SE600X vertical gel system (Hoefer Inc.; Holliston, USA), were used to electrophoretically separate nebulin from other proteins, such as titin and myosin heavy chain. The samples were run in five incremental loading volumes. Gels were run at 15 mA per gel for 3 h and 15 min, then stained using Neuhoff’s Coomassie staining protocol and scanned using a commercial scanner (Epson 800, Epson Corporation, Long Beach CA). Nebulin expression was quantified from the gel images and normalised against myosin heavy chain abundance in each lane.

### Histology and immunostaining

Extensor digitorum longus (EDL), soleus (SOL), tibialis anterior, gastrocnemius, quadriceps femoris, diaphragm, and masseter skeletal muscles were selected on the basis of their suitability for the studies and potential involvement in *NEB*-NM. The muscles were collected from mice aged 4 to 12 months and snap frozen in isopentane cooled with liquid nitrogen. Sections of 8–10 μm were cut on a Leica CM3050S cryostat, then stained with haematoxylin and eosin (H&E), Gomori trichrome or succinate dehydrogenase (SDH) using standard histochemical techniques [20].

Muscle sections of 10 μm were fixed in 2% paraformaldehyde (PFA), blocked in phosphate buffered saline (PBS) with 10% foetal calf serum (FCS; Gibco), 1% bovine serum albumin (BSA; Sigma) and 1% saponin (Sigma) for 1 h. Mouse monoclonal antibody SERCA1 ATPase (diluted 1:1000, MA3–911, ThermoFisher) was conjugated to Zenon® Alexa Fluor® 594 (Life Technologies) and incubated with phalloidin-fluorescein isothiocyanate (diluted 1:1000; P5282, Sigma), at 4 °C overnight. Phalloidin tetramethylrhodamine was used alone (diluted 1:100, P1951, Sigma). Slides were washed in PBS, counterstained in Hoechst (Sigma) and mounted in Fluoromount (Sigma). Muscle sections to be incubated with mouse monoclonal alpha-actinin (diluted 1:10, EA-53, Sigma) followed the same protocol as above, however, sections were not fixed and saponin was omitted from the blocking solution.

### Immunostaining for fibre typing

Muscle sections of 9-month-old *Neb*^Y2303H,Y935X^ and age matched wild-type littermates were labelled as described previously [54]. Briefly, after blocking, mouse IgG_1_ antibodies against myosin heavy chain MHCI (slow myosin I; diluted 1:20, NCL-MHCs, Leica Biosystems) or MHCIIA (fast myosin IIA; diluted 1:5, SC-71, DSHB) were conjugated to Zenon® Alexa Fluor® 594 (Life Technologies). Primary mouse IgM antibody MHCIIB (fast myosin IIB; diluted 1:10, BF-F3, DSHB) was then added and incubated at 4 °C overnight. The secondary antibody Zenon® Alexa Fluor® 488 anti-mouse IgM was sequentially incubated at room temperature for 60 min. Slides were washed in PBS, counterstained in Hoescht (Sigma) and mounted in Fluoromount (Sigma).

### Myofibre sizes and fibre type proportions

Fibre typing of the soleus and extensor digitorum longus muscles was conducted on merged images showing (1) MHCI with MHCIIA, and (2) MHCIIA with MHCIIB (see immunostaining for methods). Fibres of each different type were counted and the Feret’s diameter measured using ImageJ software (various versions; US National Institute of Health, USA).

### Electron microscopy

Extensor digitorum longus, soleus, tibialis anterior, gastrocnemius, quadriceps femoris and masseter skeletal muscles from 4 to 9-month-old *Neb*^Y2303H,Y935X^ and age matched wild-type littermates were prepared for electron microscopy to assess their ultrastructure. After excision, the muscle was immersed in 2.5% phosphate buffered glutaraldehyde. Tissue was cut into approximately 2 × 1 mm strips and processed using a Leica tissue processor, immersed in 1% aqueous osmium tetroxide, graduated ethanols, propylene oxide, followed by araldite resin. Blocks were polymerised in a 70 °C oven overnight.

Thin sections were cut on an ultratome (RMC Boeckeler) and stained after drying on copper grids with saturated aqueous uranyl acetate and lead citrate according to standard techniques. The images were captured using a GATAN Orius 11 megapixel digital camera attached to a JEOL 1400 transmission electron microscope.

### Whole muscle physiology

Seven-month-old (± 6 days) male mice were anaesthetised (pentobarbitone via intraperitoneal injection, 40 mg/kg body weight) and the extensor digitorum longus (EDL) and soleus (SOL) surgically excised and mounted onto an in vitro muscle test system (model 1205A; Aurora Scientific Inc.). Muscles were maintained in an organ bath filled with Krebs mammalian ringer solution (121 mM NaCl, 5.4 mM KCl, 1.2 mM MgSO_4_.7H_2_O, 25 mM NaHCO_3_, 5 mM HEPES, 11.5 mM glucose and 2.5 mM CaCl_2_, pH 7.3), bubbled with carbogen (5% CO_2_ in O_2_) and maintained at 25 °C [7].

Isolated muscles were manually adjusted to the optimal muscle length (L_o_), which was determined as the muscle length that produced maximum twitch force. The twitch time-course was quantitated by measuring contraction time (time-to-peak), maximum rate of force development (max dF/dt) and half-relaxation time. The force-stimulation frequency relationship was established by exposing muscles to stimulation frequencies of 10, 20, 30, 40, 60, 70, 80, 100, 120, 150 and 200 Hz (EDL), or 5, 10, 15, 20, 30, 40, 60, 80, 100 and 120 Hz (SOL). Muscles were given a 2 min rest period beween stimulations to prevent fatigue affecting force output. The susceptibility of muscles to eccentric damage was determined by exposing muscles to five sequential eccentric contractions, where muscles were stimulated maximally (EDL, 120 Hz; SOL, 80 Hz) for 1 s while being stretched to 105, 110, 120, 130 and 140% of optimal myofibre length at a constant velocity of 1 L_o_s^− 1^. In each eccentric contraction, an initial isometric contraction was induced, and when isometric force had plateaued, the muscle was stretched. A brief, transient increase in force peak occurs at the onset of the stretch, and the height of this transient response was measured (peak stretch-induced force minus isometric plateau force) to provide information about the stiffness of the muscle, with stiffer muscle preparations producing higher transient force peaks. The amplitude of the transient stretch-related force response was normalised to the amplitude of the preceeding isometric phase of the contraction (% of isometric plateau force amplitude) [68]. In order to determine the force deficit occurring due to eccentric contraction-induced damage, a maximal isometric contraction was performed before and after each eccentric contraction and the amplitude of these maximal contractions was compared [41].

At the end of the experiment, muscles were removed from the organ bath, stripped of tendons, blotted and weighed. Muscle cross-sectional area (CSA) was determined by dividing muscle wet mass (mg) by the product of the optimal myofibre length and mammalian skeletal muscle density (1.056 mg/mm^3^) [51]. Optimal myofibre length was calculated by multiplying L_o_ by a pre-determined myofibre to muscle length ratio for the EDL (0.44) and SOL (0.71) [11]. The specific force (force normalised to muscle cross-sectional area, N/cm^2^) was calculated by dividing isometric force by the CSA.

### Single myofibre physiology

#### Myofibre permeabilisation

Relaxing and activating solutions contained 4 mM Mg-ATP, 1 mM free Mg^2+^, 20 mM imidazole, 7 mM EGTA, 14.5 mM creatine phosphate, and KCl to adjust the ionic strength to 180 mM and pH to 7.0*.* The concentrations of free Ca^2+^ were 10^–9.00^ M (relaxing solution) and 10^–4.50^ M (activating solution).

After excision, the tibialis anterior muscles from 6-month-old male mice were placed in relaxing solution at 4 °C. Bundles of ~ 50 myofibres were dissected free and tied with surgical silk to glass capillary tubes at slightly stretched lengths. Bundles were treated with skinning solution (relaxing solution with glycerol; 50:50 v/v) for 24 h at 4 °C, and transferred to − 20 °C. For long-term storage muscle bundles were treated with sucrose, a cryoprotectant, and within 1–2 weeks detached from the capillary tubes, snap frozen in liquid nitrogen-chilled isopentane and stored at − 80 °C [24].

#### Single myofibre force mechanics

On the day of experiment, bundles were de-sucrosed, transferred to a relaxing solution, and single myofibres dissected. Myofibres were individually attached between connectors leading to a force transducer (model 400A; Aurora Scientific) and a lever arm system (model 308B; Aurora Scientific). Sarcomere length was set to ~ 2.50 μm and the temperature to 15 °C [48, 49, 60]. Myofibre CSA was approximated from width and depth measures, assuming an elliptical circumference. Absolute maximal isometric force generation was calculated as the difference between the total tension in the activating solution (pCa 4.50) and the resting tension measured for the same myofibre in relaxing solution. Specific force was defined as absolute force divided by CSA.

Apparent rate constants of force redevelopment (*k*_tr_) were measured using a mechanical slack-restretch manoeuver. Briefly, each myofibre was transferred from relaxing solution to activating solution and allowed to generate steady-state force. The myofibre was then rapidly slackened (within 1–2 ms) by 20% of its original length, resulting in a rapid reduction of force to near zero. This was followed by a brief period of unloaded shortening (20 ms) before rapidly re-stretching to its original length [10]. *k*_tr_ was approximated by linear transformation of the half-time of force redevelopment (*k*_tr_ = 0.693/t_1/2_) as described previously [70].

Maximum unloaded shortening velocity (V_0_) was also calculated by the slack test. Once steady-state isometric force was reached, nine slacks of various amplitudes were rapidly introduced (within 1–2 ms) at one end of the myofibre [22]. Slacks were applied at different amplitudes ranging from 7 to 13% of the myofibre length [58, 59]. The myofibre was re-extended between releases (while relaxed) in order to minimize changes to sarcomere length. The time required to take up the imposed release was measured from the onset of the length step to the beginning of the tension redevelopment. A straight line was fitted to a plot of release length versus time, using least-squares regression from at least four data points. V_0_ for each myofibre segment was calculated by dividing the line slope by the myofibre segment length [22].

#### Myofilament measurements

On the day of experimentation, single myofibres were de-sucrosed, and individually dissected. Arrays of approximately nine myofibres were prepared at room temperature (RT) and used to measure thin and thick filament lengths. For each myofibre, both ends were clamped to half-split copper meshes designed for electron microscopy (SPI G100 2010C-XA, width, 3 mm) that were glued to cover slips (Menzel-Gläser, 22 × 50 mm, thickness 0.13–0.16 mm).

Myofibres were fixed in 4% PFA and permeabilised with 0.1% Triton X-100 in PBS. Tissues were blocked in 10% goat serum/PBS before incubation with primary antibodies diluted in goat serum blocking solution. Tropomodulin 4 (Tmod4) was detected using a rabbit IgG anti-TMOD4 antibody (1:100; R3577bl3c [26]) and α-actinin with a mouse IgG_1_ anti-α-actinin antibody (1:500; Clone EA-53, Abcam). For thin filament length measurements, myofibres were treated with Alexa 594-conjugated phalloidin (1:100, Molecular Probes). For detection, tissues were incubated with Alexa 488- or 594-conjugated secondary antibodies/PBS (1:1000; Molecular Probes).

Images were collected using a CellVoyager™ (CV1000) Confocal Scanner Box microscope using a 60x oil objective lens. The CV100 software was used for image collection and the myofibres were analysed using Distributed Deconvolution (DDecon) [56].

### Statistical analyses

The unpaired Mann-Whitney, unpaired t-test, and Welch’s t-test or two-way ANOVA were used for statistical comparison of datasets, with *p*-values < 0.05 considered to be statistically significant. Fibre typing and whole muscle physiology data are presented as mean ± SEM, and the rest of the data are presented as mean ± SD.

## Results

### *Neb*^Y2303H,Y935X^ mice do not have an apparent shorter lifespan than wild-type mice

Compound heterozygous mice (*Neb*^Y2303H,Y935X^) were produced at an occurrence of approximately one in eight offspring, as expected by Mendelian ratios. Limited numbers of *Neb*^Y935X(+/+)^ mice were born, well below proportions expected by Mendelian ratios, and mice with this genotype all died by 5 days after birth. The lifespan of *Neb*^Y2303H^ and *Neb*^Y2303H,Y935X^ mice was not overtly reduced, as all survived past the oldest time point in this study (12 months), unless sacrificed earlier.

### *Neb*^Y2303H,Y935X^ mice express the p.Tyr2303His *Neb* allele at ~ 50% of normal RNA levels, while the p.Tyr935* transcript is not detected (Fig. 1a-c)

**Fig. 1:**
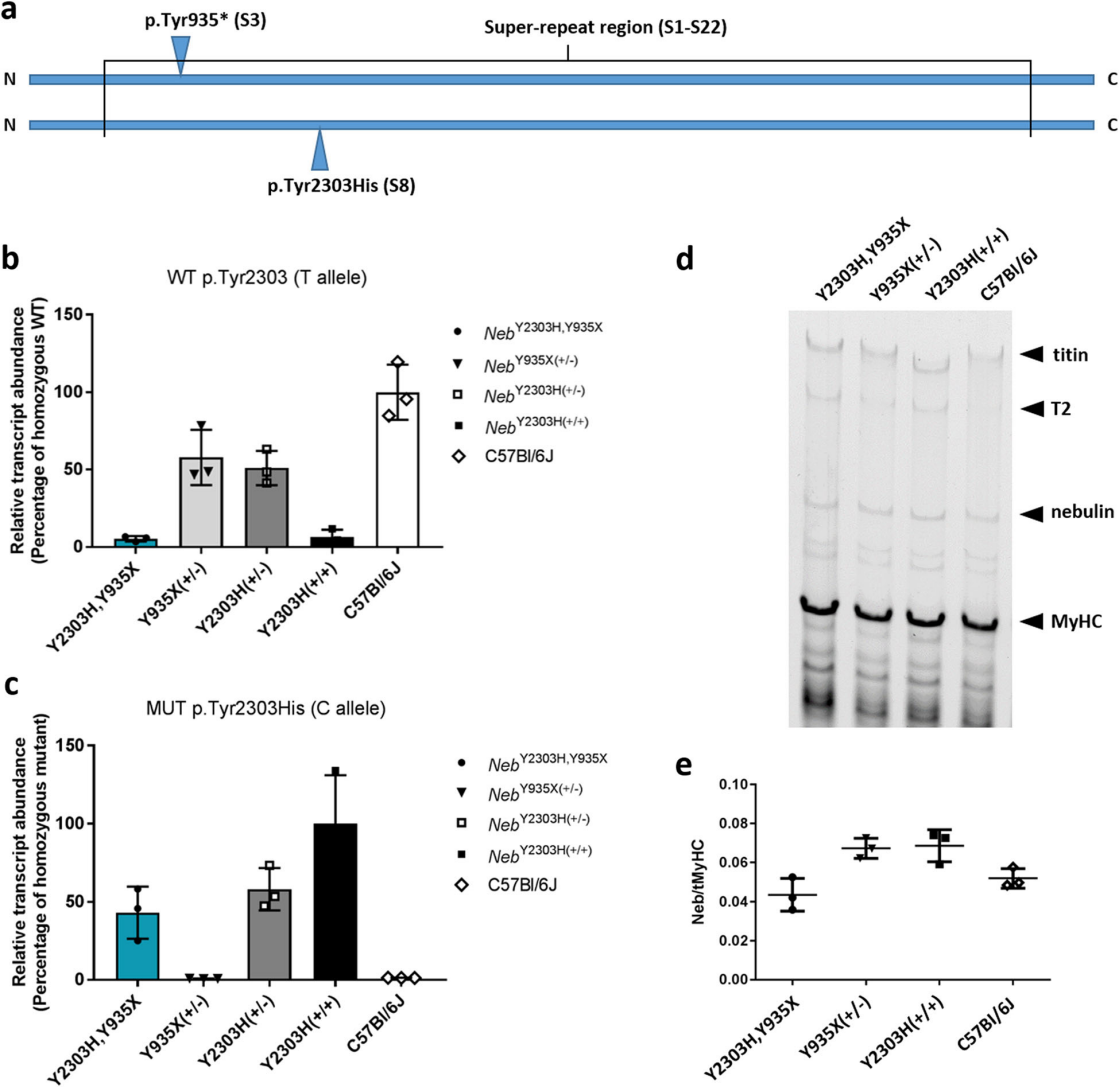
*Neb*^Y2303H,Y935X^ mice express 50% of the Tyr2303His missense allele, while the Tyr935* nonsense transcript is not detected. **a** A schematic representation of the location of the selected mutations on the nebulin protein. Primers, which differentiate between the (**b**) wild type (WT; Tyr2303, T allele) and (**c**) missense (MUT; Tyr2303His, C allele) transcripts were used to generate allele-specific qPCRs. Relative *Neb* expression was determined using the delta Ct method and normalised to the geometric mean of two endogenous control genes, *Tbp* and *Eef2.* Expression of *Neb* transcript from *Neb*^Y2303H,Y935X^ mice was compared with expression from the parental lines (*Neb*^Y2303H(+/+)^, *Neb*^Y2303H(+/−)^ and *Neb*^Y935X(+/−)^) and the C57BL/6J background strain. As expected, *Neb*^Y2303H,Y935X^ mice expressed approximately 50% of the mutant allele p.Tyr2303His compared with homozygous *Neb*^Y2303H(+/+)^ mice, and no clear expression was detected of the WT Tyr2303 allele, supporting the hypothesis that the Tyr935* transcript (carrying the Tyr2303 WT allele) is lost due to nonsense-mediate mRNA decay. The low level of WT transcript detected in *Neb*^Y2303H,Y935X^ and *Neb*^Y2303H(+/+)^ samples is likely due to background amplification from the mutant allele since the WT and mutant alleles differ by only one base-pair. **d** Representative image of the protein analysis on a 1% SDS agarose gel across the mouse strains, and **e** quantification of nebulin protein, normalised to myosin heavy chain (MyHC). Nebulin protein levels remained comparable across genotypes, suggesting a compensational mechanism in the *Neb*^Y2303H,Y935X^ and *Neb*^Y935X(+/−)^ mice from the transcript not undergoing nonsense-mediated decay. Unpaired Mann-Whitney, *n* = 3, ns, *p* > 0.05

In order to ascertain the effects of the p.Tyr935* and p.Tyr2303His variants (Fig. 1a) on transcript abundance we performed two sets of qPCR reactions. The first qPCR was designed to detect the presence of the wild-type (WT) allele (but not amplify the p.Tyr2303His allele) and is presented as a percentage of WT levels (with C57BL/6J representing 100%). Since the primers were designed after the p.Tyr935* stop codon, full length *Neb* transcript produced from the p.Tyr935* allele (if any) would also be detected. It was hypothesised that the p.Tyr935* variant would result in nonsense-mediated mRNA decay. Accordingly, heterozygous *Neb*^Y935X(+/−)^ mice expressed *Neb* at ~ 50% of WT levels suggesting that the p.Tyr935* allele is indeed degraded (Fig. 1b). Similarly, mice that were heterozygous for the p.Tyr2303His variant (*Neb*^Y2303H(+/−)^) expressed WT *Neb* at 50% of control WT levels (Fig. 1b). Negligible off-target priming (WT) was detected in *Neb*^Y2303H,Y935X^ and *Neb*^Y2303H(+/+)^ samples (Fig. 1b), which is indicative of the specificity of this reaction as these two genotypes should not express any WT *Neb* transcript.

The second qPCR was performed with primers designed to detect only the p.Tyr2303His allele and the results are presented as a percentage relative to p.Tyr2303His homozygous samples (e.g. 100% mutant). In line with the expected consequences of the p.Tyr2303His variant, muscles from compound heterozygous *Neb*^Y2303H,Y935X^ and heterozygous *Neb*^Y2303H(+/−)^ mice expressed the mutant p.Tyr2303His allele at ~ 50% of the level observed in muscles from homozygous *Neb*^Y2303H(+/+)^ mice (Fig. 1c). Muscles from *Neb*^Y935X(+/−)^ and C57BL/6J mice produced negligible levels of the p.Tyr2303His allele, as expected, due to absence of this allele in these mice (Fig. 1c). Together these results confirm that *Neb*^Y2303H,Y935X^ mice express a reduced total abundance of *Neb* transcript, likely due to degradation of the p.Tyr935* transcript, and all *Neb* transcript produced contains the p.Tyr2303His variant.

### Nebulin protein levels remain comparable between the strains (Fig. 1d-e)

We investigated whether nebulin protein levels were reflective of the nebulin transcript results detected in C57BL/6J, *Neb*^Y2303H(+/+)^, *Neb*^Y935X(+/−)^ and compound heterozygous *Neb*^Y2303H,Y935X^ mice. Nebulin protein levels remained comparable between all four strains analysed (Fig. 1d-e).

### Nemaline bodies, core-like structures, split myofibres, internal nuclei and mitochondrial accumulations are present in some muscles from *Neb*^Y2303H,Y935X^ mice (Fig. 2)

**Fig. 2:**
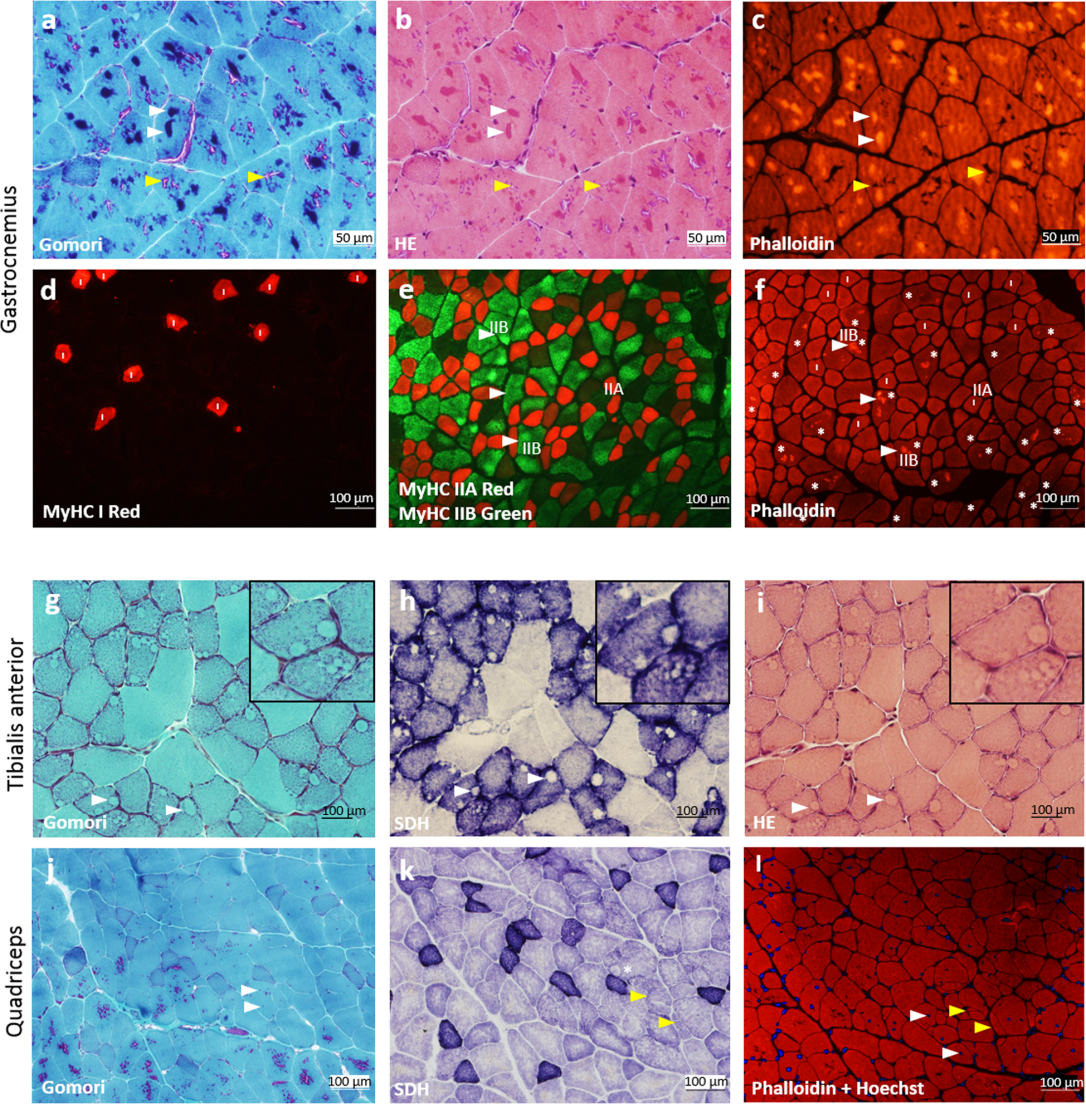
Histology and immunostaining of different skeletal muscles from *Neb*^Y2303H,Y935X^ mice demonstrate multiple pathological features. **a**-**c** Nemaline bodies (white arrowheads; purple staining in Gomori, and intense staining with TRITC-phalloidin) in serial cross-sections of gastrocnemius (9-month-old male) stained with Gomori trichrome (**a**), H&E (**b**) and TRITC-phalloidin (**c**). Tubular aggregates (yellow arrowheads; pink in Gomori, negative with phalloidin) are a non-specific finding in older male mice from certain inbred strains. **d**-**e** Fibre typing was performed on serial sections using MyHC I (**d**), and MyHC IIA and IIB (**e**), antibodies. **f** TRITC-phalloidin visualised the actin-containing nemaline bodies most commonly locating in the fast MyHC type IIB fibres. All myofibres containing definite nemaline bodies are indicated with an asterisk (*), and 25/25 of these fibres are MyHC type IIB. Nemaline bodies were occasionally found in MyHC IIA fibres, however, no nemaline bodies were found in MyHC type I (slow) fibres (all type I fibres are indicated with an “I”). **g**-**i** Cross-sections of tibialis anterior (12-month-old female) stained with Gomori trichrome (**g**), SDH (**h**), and H&E (**i**), showing core-like structures in several myofibres (white arrowheads and inset). **j**-**l** Cross-sections of quadriceps (9 month male) stained with Gomori trichrome (**j**), SDH (**k**), and TRITC-phalloidin with Hoechst (**l**) showing internal nuclei (white arrowheads), split fibres (yellow arrowheads) and an occasional core-like structure (*)

In order to determine whether muscles of *Neb*^Y2303H,Y935X^ mice exhibited similar histological features to patients with NM, a range of skeletal muscles representing different compositions of fast and slow myofibre proportions were studied. Gomori trichrome and H&E staining revealed large quantities of accumulations resembling nemaline bodies characteristic of NM disease in patients (Fig. 2a and b, white arrowheads). The accumulations were most prominent in the gastrocnemius and quadriceps of *Neb*^Y2303H,Y935X^ mice and were less abundant in the masseter, extensor digitorum longus and diaphragm at all time points studied. Interestingly, no nemaline bodies were found in soleus or tibialis anterior muscles. Phalloidin staining confirmed that the accumulations contained filamentous actin (Fig. 2c, white arrowheads), and these accumulations also stained positive for alpha-actinin. SDH staining and fibre typing with MHC antibodies indicated that the aggregates were predominantly found in MHC type IIB (fast, glycolytic) fibres (Fig. 2d-f).

Tubular aggregates were also apparent in the skeletal muscles of male mice. Although this is a non-specific finding in older inbred male mice, male *Neb*^Y2303H,Y935X^ mice at 9 months of age had abundant and abnormally large tubular aggregates, comparable to those usually seen in male C57BL/6J mice at 18 months of age [3]. Tubular aggregates were distinguished from actin-containing nemaline bodies by SERCA staining, as well as SDH and phalloidin staining, as tubular aggregates are negative for both of these markers (Fig. 2a-c).

Additional pathological features were also found in different muscles. Of the skeletal muscles studied, core-like structures were evident in the oxidative fibres of the tibialis anterior (Fig. 2g-i) and masseter muscles, and occasionally in the quadriceps (Fig. 2k). Core-like structures did not correspond to areas of nemaline body aggregation, and they stained negative with SDH staining, confirming that these areas were devoid of mitochondria. Cores were only present in a subset of fibres (fast and slow), and could not be confirmed with electron microscopy. Several split myofibres were seen in the quadriceps (Fig. 2j-l) and extensor digitorum longus. Furthermore, internal nuclei and apparent myofibre size variation were observed in the quadriceps, but due to the heterogeneity of the quadriceps muscle, these features were not further quantified (Fig. 2j-l).

**Fig. 3:**
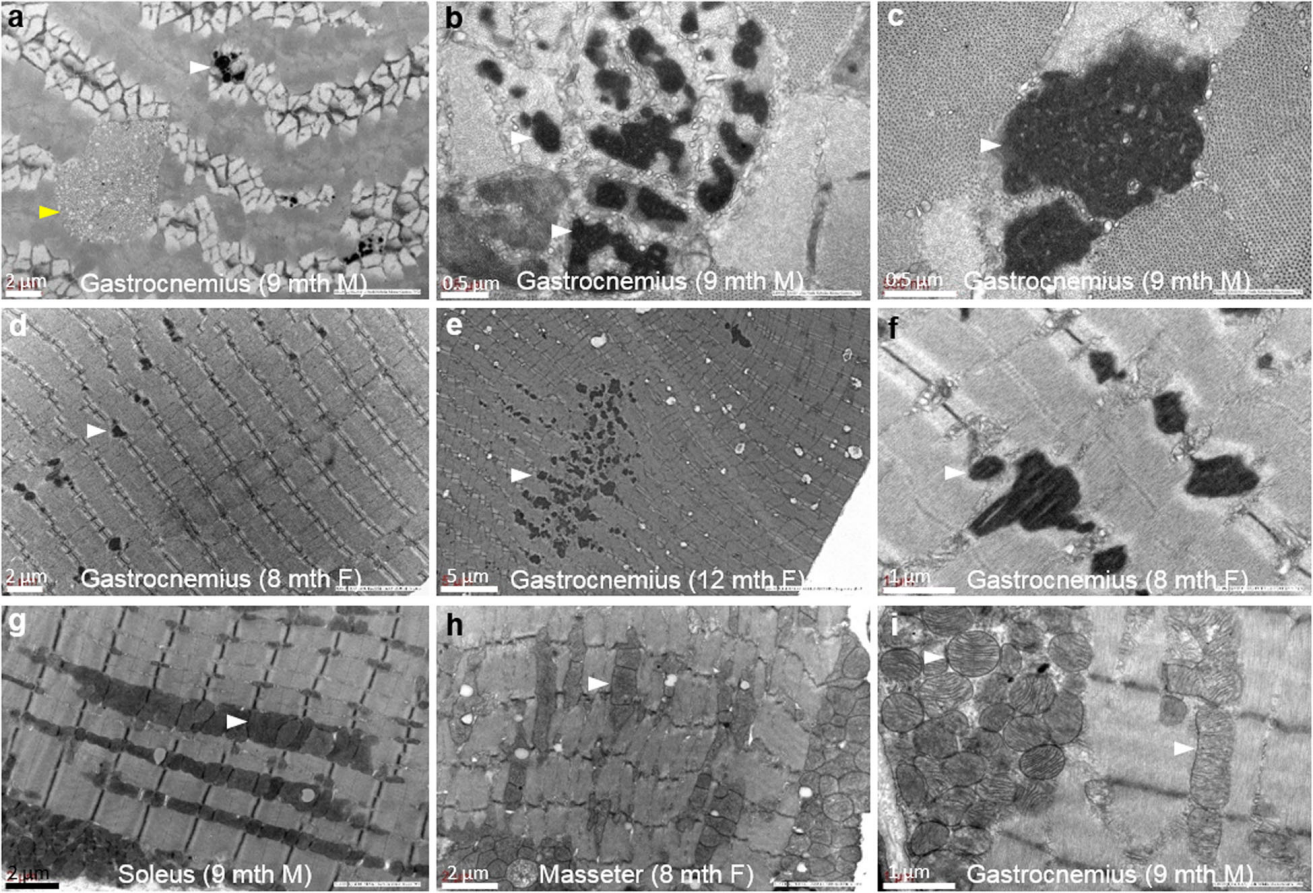
Ultrastructural analysis of muscles from *Neb*^Y2303H,Y935X^ mice confirmed nemaline bodies, tubular aggregates and pleomorphic mitochondria. **a**-**c** Electron microscopic images showing nemaline bodies (white arrowheads) and tubular aggregates (yellow arrowheads) from a 9-month-old male mouse. **d**-**f** Nemaline bodies originating from the Z disc identified in 8–12-month-old female mice. **g**-**i** Large intermyofibrillar aggregates of mitochondria were seen in all the muscles studied, with notable variation in mitochondrial size within the aggregates

Transmission electron microscopy of the gastrocnemius from *Neb*^Y2303H,Y935X^ mice validated the presence of electron-dense material indicative of nemaline bodies (Fig. 3a-f, seen in aggregates and as expansions of Z-disc structures), and tubular aggregates (Fig. 3a). The nemaline bodies were large and irregular, and in some instances clearly originated from the Z discs (thickened Z discs [20], Fig. 3d-f). Large intermyofibrillar aggregates of mitochondria were seen in a subpopulation of myofibres of all skeletal muscles studied, with variation in mitochondrial size within the aggregates (Fig. 3g-i).

### *Neb*^Y2303H,Y935X^ mice have significantly smaller myofibre diameters (Fig. 4)

**Fig. 4:**
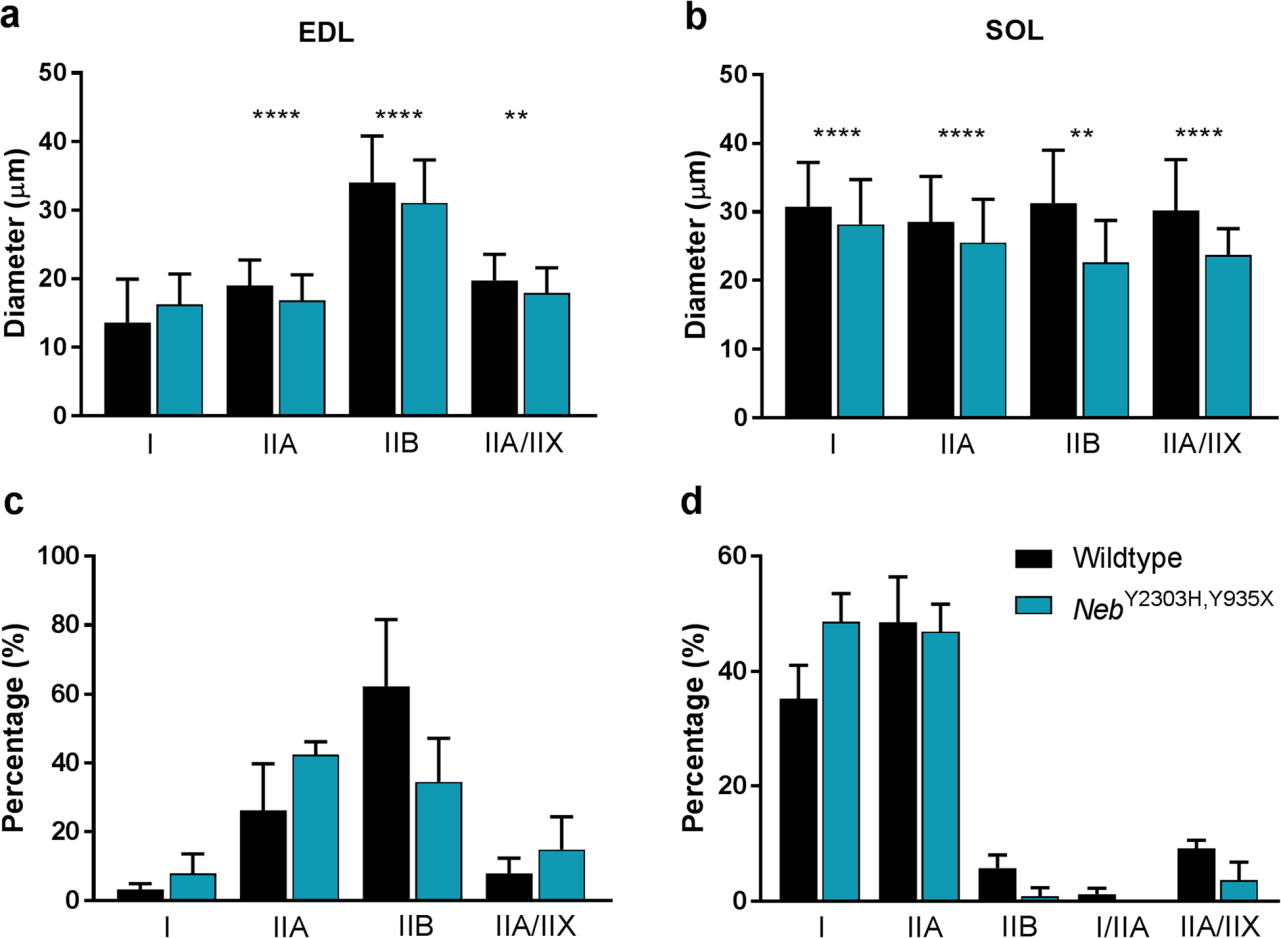
*Neb*^Y2303H,Y935X^ mice have significantly smaller myofibre diameters at 9 months of age. **a**-**d** MHC fibre types (I, IIA, IIB and mixed I/IIA and IIA/IIX) from 9-month-old female *Neb*^Y2303H,Y935X^ extensor digitorum longus (EDL) and soleus (SOL) were counted and fibre diameters measured. **a** All myofibres except MHC type I were smaller in EDL from *Neb*^Y2303H,Y935X^ mice compared with age matched wild-type littermates. **b** All fibres in SOL were smaller in *Neb*^Y2303H,Y935X^ mice, although no mixed MHC type I/IIA were identified in this cohort. **c**, **d** Fibre-type proportions were measured in EDL (**d**, *n* = 3) and SOL (**e**, *n* = 3) but were not significantly different. Unpaired Mann-Whitney, *p < 0.05; **p < 0.005; ***p < 0.0005; ****p < 0.0001

MHC fibre types (I, IIA, IIB, hybrid I/IIA and hybrid IIA/IIX) from the extensor digitorum longus (EDL) and soleus (SOL) of 9-month-old female *Neb*^Y2303H,Y935X^ mice were counted and fibre diameters measured (for the numerical data, see Additional file 1). All myofibres that contained a fast MHC (type IIA, IIB, IIA and/or IIX) were significantly smaller in the EDL from *Neb*^Y2303H,Y935X^ mice compared with age-matched wild-type littermates. However, myofibres expressing slow MHC (type I) trended towards being hypertrophied in the EDL of *Neb*^Y2303H,Y935X^ mice (Fig. 4a; type I: + 19.2%, *p* = 0.0854, ns; type IIA: − 11.3%, *p* < 0.0001; type IIB: − 6.6%, *p* < 0.0001; type IIA/IIX: − 8.9%, *p* = 0.006). All myofibres from the SOL of *Neb*^Y2303H,Y935X^ mice were significantly smaller (Fig. 4b; type I: − 8.4%, *p* < 0.0001; type IIA: − 10.6%, *p* < 0.0001, type IIB: − 27.6%, *p* = 0.0015; type IIA/IIX: − 21.7%, *p* < 0.0001).

Fibre-type proportions (as % of all myofibres) in the EDL (Fig. 4c; *n* = 3) and SOL (Fig. 4d; *n* = 3) from *Neb*^Y2303H,Y935X^ mice were trending towards being significantly different when compared with wild-type littermate controls. In both muscles the oxidative fibre types (slow MHC I or fast IIA) were more abundant (EDL, type I: + 4.68%, *p* = 0.4, ns; type IIA + 16.17%, *p* = 0.1, ns; SOL, type I: + 13.37%, *p* = 0.1, ns), whereas there were less fast twitch, glycolytic type IIB fibres (EDL, type IIB: − 27.74%, *p* = 0.1, ns; SOL, type IIB: − 4.92%, *p* = 0.1, ns). A predominance of slow, type I fibres is common in *NEB*-NM patients [86] so it is promising that our mouse model is displaying a similar trend.

### Skeletal muscles from *Neb*^Y2303H,Y935X^ mice are susceptible to eccentric contraction-induced damage (Fig. 5)

**Fig. 5:**
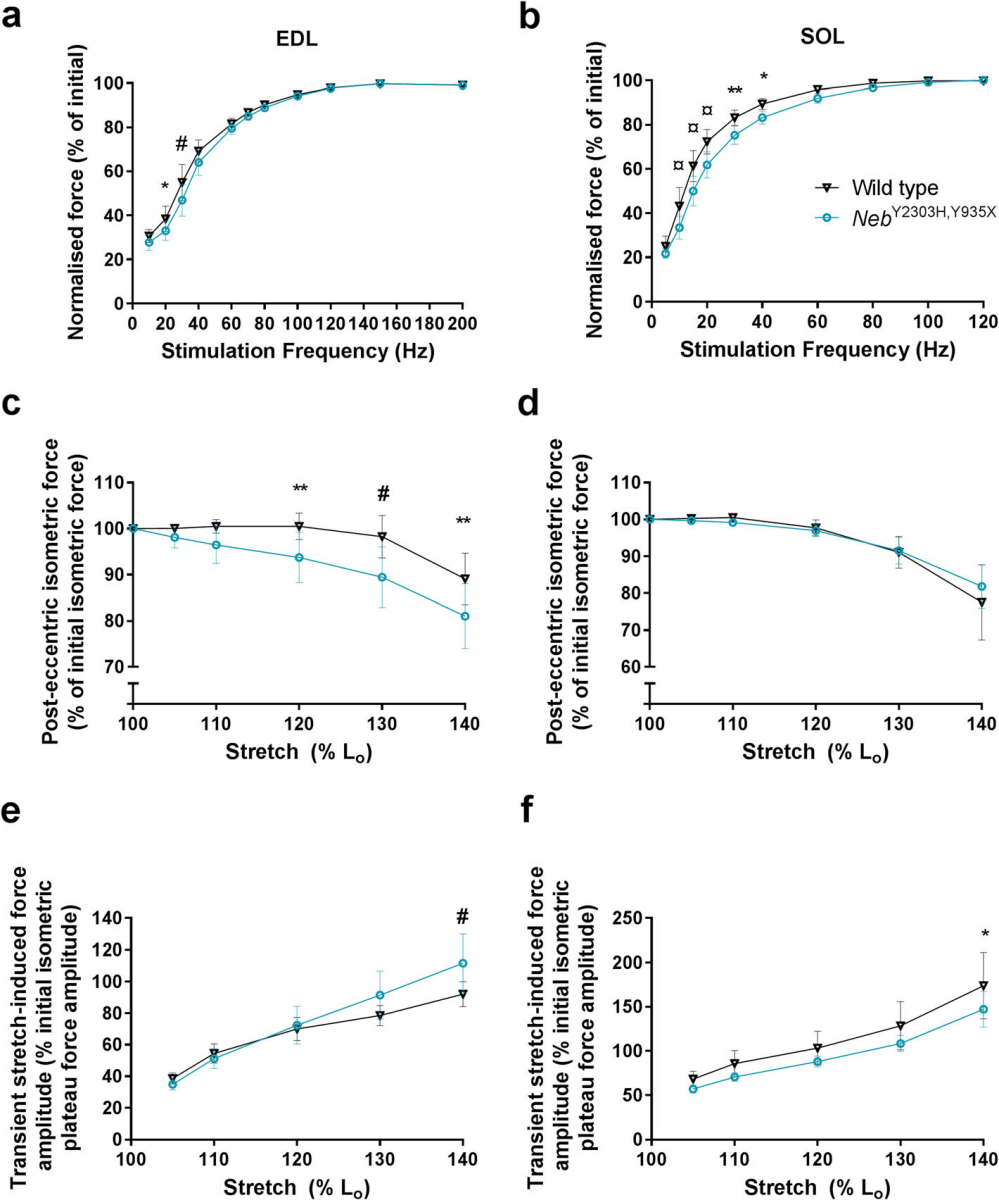
Whole-muscle physiology experiments showed the *Neb*^Y2303H,Y935X^ EDL muscle was susceptible to eccentric contraction-induced damage. **a**, **b** Whole-muscle in vitro contractile analysis of extensor digitorum longus (EDL) and soleus (SOL) from male mice at 7 months of age showed a significant deficit in normalised force production in *Neb*^Y2303H,Y935X^ at low-stimulation frequencies. **c**, **d** EDL *Neb*^Y2303H,Y935X^ muscle was more susceptible to damage by eccentric contractions involving stretches to 120 to 140% of L_o_ than wild type. **e**, **f** Transient stretch-induced force responses were higher in *Neb*^Y2303H,Y935X^ EDL muscles compared with wild type, suggesting increased stiffness in *Neb*^Y2303H,Y935X^ EDL muscles. In contrast, transient stretch-induced force responses were lower in *Neb*^Y2303H,Y935X^ SOL muscles compared with wild type, suggesting greater compliance in *Neb*^Y2303H,Y935X^ SOL muscles. *n* = 7, Two-way ANOVA, *p < 0.05; **p < 0.005; #*p* < 0.0005; ¤*p* < 0.0001

Analysis of whole muscle physiology in vitro showed twitch contraction times were faster (reduced time-to-peak values) in the extensor digitorum longus (EDL) muscles from *Neb*^Y2303H,Y935X^ mice compared with age-matched wild-type littermate controls (*p* = 0.0234). No difference in soleus (SOL) twitch contraction times was found between groups (*p* = 0.0592, ns). All other twitch parameters and maximum specific force were similar in muscles from *Neb*^Y2303H,Y935X^ and control mice (Additional file 2). However, significant decreases in normalised force were found in *Neb*^Y2303H,Y935X^ muscles compared with wild-type controls at low stimulation frequencies: EDL at 20 Hz (*p* = 0.0385) and 30 Hz (*p* = 0.0002); SOL from 10 Hz – 30 Hz (*p* < 0.0001), 40 Hz (*p* = 0.0018) and 50 Hz (*p* = 0.0307) (Fig. 5a, b). The EDL from *Neb*^Y2303H,Y935X^ mice was more susceptible to eccentric contraction-induced muscle damage than wild-type controls when stretched from 120 to 140% of L_o_ (optimal muscle length) during eccentric activation (Fig. 5c, d). According to the transient stretch-related peak force data, the EDL muscles from *Neb*^Y2303H,Y935X^ mice were significantly stiffer than those from wild-type controls (*Neb*^Y2303H,Y935X^ EDL transient stretch response was 20% greater than the wild-type response during 140% of L_o_ stretch, *p* = 0.0008). In contrast, the SOL from *Neb*^Y2303H,Y935X^ mice exhibited a significant decrease (15%) in stiffness during 140% of L_o_ stretch compared with the wild-type response (*p* = 0.0283) (Fig. 5e, f).

### Altered myosin cross-bridge kinetics potentially underlying force depression in muscles from *Neb*^Y2303H,Y935X^ mice (Fig. 6a-c)

**Fig. 6:**
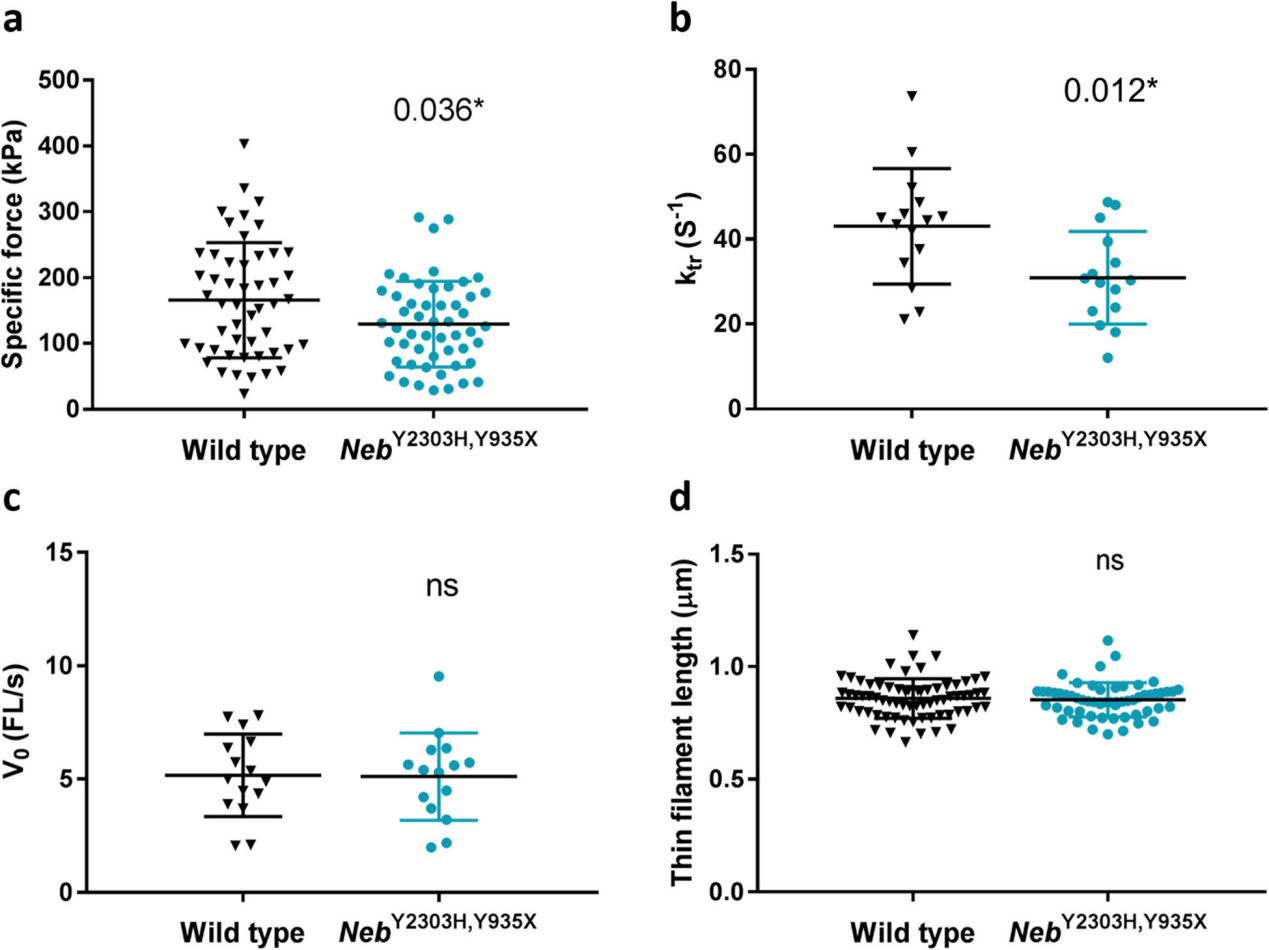
Single myofibre measurements show reduced force, slower *k*_tr_, and normal thin filament length. **a** In single-myofibre physiology measurements 6-month-old male *Neb*^Y2303H,Y935X^ mice had lower mean specific force compared with wild-type littermate controls. **b** Additionally, the mean *k*_tr_ (tension redevelopment) was slower in *Neb*^Y2303H,Y935X^ mice, however, **c** V_0_ was unaffected. These results indicate a potential alteration of myosin cross-bridge kinetics underlying the force depression. Welch’s T-test, *p < 0.05. **d** The thin filament length was not altered in *Neb*^Y2303H,Y935X^ mice

The single myofibre preparation allows direct measurements of contractility with an intact myofilament lattice without the confounding effects of nerves, excitation-contraction coupling, myofibre architecture and inter-cellular connective tissue. Tibials anterior muscles were isolated from 6-month-old male mice and the dissected myofibres (mainly IIX and IIB fibres) were used in the experiments. The mean maximum specific force was 22% lower in *Neb*^Y2303H,Y935X^ mice than in wild-type mice (Fig. 6a, *p* = 0.036). Additionally, the mean *k*_tr_ (rate of force redevelopment) was 28% slower in *Neb*^Y2303H,Y935X^ mice (Fig. 6b; *p* = 0.012). The V_0_ (maximum unloaded shortening velocity) was unaffected in *Neb*^Y2303H,Y935X^ mice (Fig. 6c). Taken together, these results indicate a potential alteration of myosin cross-bridge kinetics underlying the force depression. *k*_tr_ reflects the myosin cross-bridge cycle turnover rate and according to the two-state cross-bridge model, it is proportional to *f*_app_ + *g*_app_, with *f*_app_ being the rate constant for attachment and *g*_app_ the rate constant for detachment. V_0_ has *g*_app_ as a rate limiting step. Hence, in *Neb*^Y2303H,Y935X^ mice the combination of decreased *k*_tr_ and maintained V_0_ indicates a dramatic reduction in *f*_app_. This is likely to shorten the time spent by each myosin molecule in a strongly bound force-producing conformation limiting the fraction of active myosin cross-bridges. Note that fibres were not assessed for their myosin heavy chain composition. Hence, it is not totally excluded that some of our results may be due to fibre type differences.

### Muscles from *Neb*^Y2303H,Y935X^ mice have a preserved thin filament length (Fig. 6d)

Immunostaining of single myofibres with Tmod4 and α-actinin antibodies were used to measure sarcomeric distances. No differences in thin filament lengths were detected over a range of sarcomere lengths (Fig. 6d) in *Neb*^Y2303H,Y935X^ mice compared with wild-type mice. Alpha-actinin was correctly localised and showed regular striation patterns, indicating preserved Z-disc structures in *Neb*^Y2303H,Y935X^ mice.

### Assessment of exercise function revealed that *Neb*^Y2303H,Y935X^ mice display a mild phenotype

Female *Neb*^Y2303H,Y935X^ mice were significantly smaller than controls at 6 months, and similarly, *Neb*^Y935X (+/−)^ females weighed significantly less at both 3 months and 6 months (Additional file 3a: Six month time point). Body weights of male and female *Neb*^Y2303H^ mice collected at 3 months and 6 months of age were not significantly different to wild-type littermate controls.

Investigation of muscle function using grip strength test only showed a deficit in muscle force in 6 month female *Neb*^Y2303H,Y935X^ mice (Additional file 3b). There was no distinction in force between wild-type controls and male *Neb*^Y2303H,Y935X^ mice as was also the case for *Neb*^Y2303H^ or *Neb*^Y935X^ mice for either sex or time point analysed.

Investigating muscle function using voluntary running wheels yielded inconclusive results due to high variability in each cohort analysed. While female *Neb*^Y2303H,Y935X^ mice displayed a significantly decreased performance in daily distance, average speed and maximum speed at 6 months (Additional file 3c) these results were not observed at the 3 month time point, or in male mice at either time point. Similarly, no significant differences were seen in any parameter for male and female *Neb*^Y2303H^ or *Neb*^Y935X^ mice at either time point. Some mice were capable of running distances comparable to wild-type littermate controls while others did not run at all. Therefore, this may not be the most accurate measure of muscle function for these mouse models.

No significant differences were seen for any cohort that underwent rotarod analysis.

## Discussion

As most NM patients with *NEB* mutations have a compound heterozygous genotype and do not have a severe phenotype, there is a need for an animal model that accurately represents these features. The only murine model to date that has harboured a mutation akin to those found in human patients has been the *Neb*^Δexon55^ mouse [61] with homozgyous deletion of exon 55. However the *Neb*^Δexon55^ model had a very severe NM phenotype, with mice exhibiting dramatic growth retardation and death occurring within the first week of life. This phenotype was very different from the observed phenotype of patients with the equivalent homozygous exon 55 deletion [43]. Contrastingly, another murine model with a large deletion of the C-terminus of nebulin, *Neb*^ΔSH3^, had no observable disease phenotype [90]. The *Neb*^Δ163–166^ mouse that lacks both the C-terminal domains, SRC homology 3 domain (SH3) and serine rich region (SRR), had a moderate myopathic phenotype [46]. All other published *Neb* murine models have been knock-outs (KO) of *Neb* [8, 47, 89] and are not genetically appropriate models for investigation, as a complete absence of nebulin expression has never been identified in a *NEB*-NM patient [44]. Muscle defects in mice often result in no abnormal phenotype or less severe clinical phenotypes than in humans [15], which complicates developing a mouse model with a milder phenotype. However, to effectively study nebulin function and the pathogenesis of *NEB*-NM, a model with a longer lifespan is needed. To this end, we have characterised a murine model with a compound heterozygous *Neb* mutation genotype.

*Neb*^Y2303H,Y935X^ mice survive to adulthood and in the current study were all sacrificed by 1 year of age, which is equal to approximately 40 years in humans [21]. This finding aligns with the knowledge that most patients with milder forms of NM do not have an obviously shortened life expectancy [73]. NM patients with two truncating, i.e. frameshift or nonsense mutations in constitutively spliced exons 5′ of exon 180 have not been identified, suggesting that a complete loss of nebulin is not compatible with human life. In the case of two truncating mutations being present, either both or at least one is in an alternatively spliced exon [44]. The *Neb*^Y935X(+/+)^ mice, with two loss-of-function alleles, are expected to have complete loss of the nebulin protein, and, indeed, the phenotype is early lethal. This is also in line with previous studies using the *Neb*-KO models [8, 89]. The total *Neb* transcript level was close to normal in the muscles of heterozygous *Neb*-KO mice [25], despite genetically only having 50% of *Neb*. Furthermore, no differences in total protein levels were detected in heterozygous *Neb*-KO mice, suggesting a compensation mechanism in the wild-type nebulin expression. Only 50% of nebulin RNA was expressed in *Neb*^Y2303H,Y935X^ mice and all of these transcripts were expected to contain the missense mutation. Expression of low levels of truncated proteins from transcripts escaping the nonsense-mediated decay pathway cannot, however, be excluded. In rare autosomal dominant cases, a truncated nebulin protein seems to act in a dominant-negative way, contributing to the disease phenotype [33]. Despite the lower level of transcript expressed, the total nebulin protein levels were not found to be decreased in the novel mice studied, indicating a compensation at the protein level from the transcript expressed. Lower levels of nebulin have been seen in some, but not all, NM patients and mice [42, 57, 61, 63], suggesting that a reduction in nebulin protein level is not always associated with the NM phenotype. The differences in nebulin levels across NM patients and mouse models indicate another potential pathogenetic mechanism, i.e. the lower protein level may also contribute to the cascade of events leading to NM. As the *Neb*^Y2303H^ mice, with homozygous missense mutations, had no clear disease phenotype, a currently unknown additional mechanism must play a role in the pathogenesis of the NM phenotype in the compound heterozygous model.

The missense variant changes a perfectly conserved amino acid (p.Tyr2303His) in one of the known canonical actin-binding sites (SDxxYK) in super repeat eight (S8), which in human nebulin is known to bind actin weakly [39]. It has been hypothesised that a missense change in an actin-binding site is potentially pathogenic [44], and that a mismatch between nebulin and actin may increase susceptibility to proteolysis [62]. It is also possible that a missense variant in a weakly binding repeat could strengthen actin binding, thus disrupting the dynamic movement of the thin filament proteins in muscle contraction. In compound heterozygous NM patients a missense variant in *NEB* is usually coupled with another, more disruptive mutation [44], as is the case with the *Neb*^Y2303H,Y935X^ mice. The exact missense change p.Tyr2303His (corresponding to p.Tyr2308His in the human protein, NP_001258137.1) has not been described in patients. However, the tyrosine in question is 100% conserved, not only across all of the over 200 actin-binding sites in nebulin, but also across species, highlighting its importance. According to our records, there are six cases with a combination of a missense variant affecting the tyrosine in another conserved actin-binding site, coupled with a nonsense, frameshift or a splice site change in the other allele [44]. Five out of six of these patients presented with typical NM, and one out of six with a mild form of NM. In homozygous form, missense variants lead to a different disease entity, distal nebulin myopathy [84]. As many variant combinations are unique to NM families, genotype-phenotype correlations are difficult to establish. To study disease pathogenesis, a model with a combination of a missense and nonsense mutation is ideal for representing the mild to moderate NM phenotype.

Nemaline bodies are the defining pathological feature in the skeletal muscles of NM patients, regardless of genetic cause, although their abundance does not correlate with disease severity [6, 16, 72]. The skeletal muscles of *Neb*^Y2303H,Y935X^ mice exhibit nemaline bodies, thereby confirming that they are a mouse model of *NEB-*NM disease. Nemaline bodies were present in *Neb*^Y2303H,Y935X^ mouse muscles at the age of 4 months, which is the earliest time point studied histologically. Proteins originating from the thin filament or Z disc are known components of nemaline bodies [86], which is consistent with the presence of filamentous actin and alpha-actinin in the nemaline bodies found in *Neb*^Y2303H,Y935X^ mice. Variance in distribution of nemaline bodies between skeletal muscles is often seen in *NEB*-NM patients [78], and they are most abundant in diaphragm, tongue and masseter [23, 35, 50, 53, 79, 82]. Nemaline bodies in *NEB*-NM patients are found in both fast and slow myofibres, but they may only be present in a limited area of the sample [78, 86]. Similarly, in *Neb*^Y2303H,Y935X^ mice, the aggregates were not evenly distributed within skeletal muscles. In contrast to the majority of *NEB*-NM patients, nemaline bodies in *Neb*^Y2303H,Y935X^ mice were preferentially localised in fast, glycolytic fibres (especially in MHCIIB fibres, not found in human limb muscle), whereas no nemaline bodies were found in slow myofibres. This may explain the lower abundance of nemaline bodies in the *Neb*^Y2303H,Y935X^ muscles with fewer glycolytic myofibres, i.e. soleus and diaphragm. However, it does not explain the absence in the tibialis anterior with the same fibre-type proportions as the extensor digitorum longus (MHCIIB make up 70% of total muscle fibres [90]). The reason for these differences between *Neb*^Y2303H,Y935X^ mice and *NEB*-NM patients remains to be elucidated. Skeletal muscle biopsies from *NEB*-NM patients often show type I myofibre atrophy or hypotrophy, combined with type I fibre predominance, and type II hypertrophy is occasionally observed [78]. Rare cases with type II atrophy have also been encountered [86]. Despite not being significantly different, *Neb*^Y2303H,Y935X^ mice showed a trend towards type I fibre predominance in soleus. The same trend towards more oxidative myofibre types in the soleus was also observed in the conditional *Neb*-KO mouse model [47]. MHCIIA and IIB fibres were significantly smaller, in contrast to most human nemaline patients. Additional signs of skeletal muscle damage in *Neb*^Y2303H,Y935X^ mice included the presence of split myofibres, internal nuclei and occasional fatty infiltration in quadriceps muscles. This is similar to that seen in NM patients over time [69]. A small number of patients with *NEB* mutations have been reported to have cores within their skeletal muscles, with some having a combination of a greater number of cores and nemaline bodies (and hence their disorder becoming known as “core-rod myopathy” [86]). Cores have also been identified in distal NM with *NEB* mutations [76], and in NM patients with *RYR1*, *KBTBD13*, *CFL2* [86] and *ACTA1* mutations [30].

Muscle defects in mice often result in no abnormal phenotype or less severe clinical phenotypes than in humans [2, 9, 17, 28]. Several factors may contribute to this, e.g. differences in body mass between human and mouse, bipedal versus quadrupedal movement, the resilient nature of mice, or other factors that differ between mice and humans. A thorough investigation of the in vivo phenotype revealed only minor differences between the mouse strains studied. Indeed, most of the results in the exercise tests remained comparable with wild type, and large sample sizes were required to reach the occasional significance. Overall, the results of the exercise performance were too mild and variable to be used as a reliable measure of the disease phenotype. However, the whole muscle experiments in vitro revealed that the extensor digitorum longus and soleus muscles displayed significant rightward shifts of their force-frequency relationships at lower stimulation frequencies, which is indicative of reduced Ca^2+^ sensitivity. This has been reported in muscle from patients with NM, including those with *NEB*-NM [62]. Extensor digitorum longus from *Neb*^ΔSH3^ mice also displayed a reduced relative force at lower stimulation frequencies in vitro [90]. Similarly to the *Neb*^Y2303H,Y935X^ mice, this mouse model had no visible phenotype in vivo, despite lacking the entire C-terminal SH3 domain of nebulin, which has been thought to anchor nebulin to the Z disc, among other roles. In contrast to the *Neb*^Y2303H,Y935X^ mice, the *Neb*^ΔSH3^ mice exhibited no histological or ultrastructural changes. As the *Neb*^Y2303H,Y935X^ mice recapitulate several aspects of human *NEB*-NM, it is an important model for studying nebulin function and disease pathogenesis, despite the mild clinical phenotype.

Whole muscle physiological studies on *Neb*^Y2303H,Y935X^ mice indicated increased susceptibility to contraction-induced damage, which potentially occurs when a muscle is stretched as it is contracting (e.g. when people walk downhill). This has also been reported in *Neb*^ΔSH3^ mice [90]. Additionally, we found evidence indicating a decrease in stiffness in the soleus muscles from *Neb*^Y2303H,Y935X^ mice compared with wild-type mice, which is consistent with recent reports showing that stiffness is reduced in slow muscles from nebulin knock-out mice [31]. In contrast to this, our results also showed that extensor digitorum longus muscles from *Neb*^Y2303H,Y935X^ mice are significantly stiffer than those of wild-type mice. This difference in the effects of the combination of the *Neb*^Y2303H^ and *Neb*^Y935X^ variants in fast and slow muscles could be due to differences in the effects of one or both variants on the function of the shorter nebulin isoform found in fast myofibres [67]. An increase in stiffness can also be associated with splitting of myofibres (as often seen over time in NM patients [85]), and were occasionally seen in the extensor digitorum longus and quadriceps of *Neb*^Y2303H,Y935X^ mice. This may suggest that the increased stiffness occurs predominantly in the fast twitch muscles, as no split myofibres were identified in the soleus. The missense change p.Tyr2303His could potentially affect the interaction between nebulin and actin, resulting in the increased stiffness observed in the extensor digitorum longus muscle of the *Neb*^Y2303H,Y935X^ mice. Further studies are needed, however, to elucidate the exact mechanism by which stiffness is increased.

Shortened thin filaments have been seen in several of the previous *Neb* mouse models [8, 47, 61, 89], and in some, but not all *NEB*-NM patients [87], leading to the hypothesis that the reduction is mutation specific [57, 87]. Shortened thin filaments are thought to contribute to the force deficit observed in the corresponding mouse models [47]. However, as some patients with mutations in *NEB* have displayed significant force deficits with normal thin filament lengths, other mechanisms must also affect force production [87]. Similarly, single myofibres from *Neb*^Y2303H,Y935X^ tibialis anterior muscles had lower maximum force production, yet no change in thin filament length. No difference in maximum force was detected at a whole muscle level (extensor digitorum longus and soleus) for *Neb*^Y2303H,Y935X^ mice, and thus it is likely that the calcium transient and/or the muscle architecture (e.g. pennation angle, quantity of non-contractile material) are recompensing for the force deficit detected at the myofilament level.

Taken together, our data suggest that the nebulin defects harboured by these mice alter myosin binding to actin (potentially a slower attachment rate), thus disrupting cross-bridge cycling and ultimately perturbing force production. Altered myosin cross-bridge kinetics has frequently been found to underly force depression in NM models [88]. Force generated per cross-bridge, and the number of strongly bound cross-bridges both contribute to the force generated at a given overlap between the filaments. Cross-bridge cycling kinetics determine both of these quantifiers by modulating the time spent in the strongly bound state. Chandra and co-workers found that nebulin does not affect the force produced per individual cross-bridge in the *Neb*-KO mouse model [12]. Our results corroborate this, as decreased time spent by individual myosin molecules in a strongly attached force-producing conformation was observed in the *Neb*^Y2303H,Y935X^ mice. These physiological attributes detected in skeletal muscles of *Neb*^Y2303H,Y935X^ align with previous measurements of samples from *NEB*-NM patients [57].

## Conclusions

Characterisation of phenotypic aspects of *Neb*^Y2303H,Y935X^ mice with compound heterozygous *Neb* mutations, like most *NEB*-NM mutations, has determined that they are a suitable murine model of *NEB*-NM. They exhibit nemaline bodies within their skeletal muscles and have several other histological and physiological parameters resembling NM. These findings make *Neb*^Y2303H,Y935X^ mice the most appropriate mouse model of *NEB*-NM thus far. Despite the mild in vivo phenotype, the *Neb*^Y2303H,Y935X^ mice, along with their corresponding parental lines that carry either the missense or the nonsense mutation, will be useful in deciphering nebulin function and the pathogenetic mechanisms of *NEB*-NM. Additionally, they may constitute a good animal model for primary myosin motor dysfunction, and are likely to be valuable for the assessment of potential therapeutic approaches for *NEB*-NM.

## Supplementary information

**Additional file 1. **Fibre sizes and fibre-type proportions as numbers. 9-month-old female *Neb*^Y2303H,Y935X^ mice, mean with standard deviation. Unpaired Mann-Whitney, *n* = 3, *p < 0.05; **p < 0.005; ***p < 0.0005; ****p < 0.0001.

**Additional file 2. **Summary of the whole muscle physiology results. **a** Individual muscle mass, maximum specific force, twitch specific force, maximal rate of force production (dF/dt), contraction time (time-to-peak), half-relaxation time. Unpaired t-test. **b** Force frequency, **c** eccentric damage, **d** eccentric peak stretch. Two-way ANOVA (Sidak’s multiple comparisons test). Data presented as mean +/− SEM; *n* = 8; *p < 0.05; **p < 0.005; ***p < 0.0005; ****p < 0.0001.

**Additional file 3. **Bodyweight and gross functional changes of *Neb*^Y2303H,Y935X^, *Neb*^Y935X^ and *Neb*^Y2303H^ mice at 6 months of age. **a** Female *Neb*^Y2303H,Y935X^ and *Neb*^Y935X^ mice weighed less (*p* = 0.017* and *p* = 0.014*, respectively) than wild-type littermate controls. The functional performance of the mice was tested using whole animal in vivo experiments. **b** Grip strength testing showed reduced average grip (*p* = 0.013*) and reduced maximum grip (*p* = 0.0039**) for the female *Neb*^Y2303H,Y935X^ mice. **c** Female *Neb*^Y2303H,Y935X^ mice had impaired performance for three of the four voluntary running wheel measures; distance (*p* = 0.04*), max speed (*p* = 0.026*), duration (*p* = 0.072), and average speed (*p* = 0.038*). However, no significant differences were demonstrated with the rotarod analyses (data not shown), or for the males in any of the functional tests. Mann-Whitney, *p < 0.05; **p < 0.005, *n* = 10–22 for each parameter.

## Data Availability

All data generated or analysed during this study are included in this published article and its supplementary information files.

## Abbreviations

BSA: Bovine serum albumin
CSA: Cross-sectional area
dF/dt: Maximum rate of force development
EDL: Extensor digitorum longus
ENU: N-ethyl-N-nitrosourea
*f*_app_: Rate constant for (myosin) attachment
FCS: Foetal calf serum
*g*_app_: Rate constant for (myosin) detachment
H&E: Haematoxylin and eosin
kb: Kilobase
kDa: Kilodalton
KO: Knock-out
*k*_tr_: Rate of force redevelopment
L_o_: Optimal muscle length
MHCI: Myosin heavy chain I (slow)
MHCII: Myosin heavy chain II (fast)
*NEB*: Human nebulin gene
*Neb*: Mouse nebulin gene
*NEB*-NM: Nebulin related nemaline myopathy
NM: Nemaline myopathy
PBS: Phosphate buffered saline
PFA: Paraformaldehyde
qRT-PCR: Quantitative reverse-transcriptase PCR
RT: Room temperature
SDH: Succinate dehydrogenase
SH3: SRC homology 3 domain
SOL: Soleus
SRR: Serine rich region
TA: Tibialis anterior
Tmod: Tropomodulin
V_0_: Maximum unloaded shortening velocity
WT: Wild type
