# Supplementary material for: Nebulin nemaline myopathy recapitulated in a compound heterozygous mouse model with both a missense and a nonsense mutation in Neb
Source: Acta Neuropathol Commun. 2020 Feb 17;8:18. doi: 10.1186/s40478-020-0893-1 (PMC7027239; doi:10.1186/s40478-020-0893-1)
Supplement: Supplementary file 1 — Additional file 1. Fibre sizes and fibre-type proportions as numbers. 9-month-old female NebY2303H,Y935X mice, mean with standard deviation. Unpaired Mann-Whitney, n = 3, *p < 0.05; **p < 0.005; ***p < 0.0005; ****p < 0.0001. [file 40478_2020_893_MOESM1_ESM.docx]

**Additional file 1. Fibre sizes and fibre-type proportions.** 9-month-old female *Neb*^Y2303H,Y935X^ mice, mean with standard deviation. n=3, unpaired Mann-Whitney, n=3, *p < 0.05; **p < 0.005; ***p < 0.0005; ****p < 0.0001

|  | **EDL** | | | | | | | **SOL** | | | | | | |
| --- | --- | --- | --- | --- | --- | --- | --- | --- | --- | --- | --- | --- | --- | --- |
|  | **Wild type** | | ***Neb*^Y2303H,Y935X^** | | | | | **Wild type** | | ***Neb*^Y2303H,Y935X^** | | | | |
| **Diameter µM** | **Mean** | **SD** | **Mean** | **SD** | **(+/-) %** | **p** | | **Mean** | **SD** | **Mean** | **SD** | **(+/-) %** | **p** | |
| Type I | 13.65 | 6.3 | 16.27 | 4.4 | +19.2 | 0.0854 | ns | 30.75 | 6.5 | 28.18 | 6.5 | -8.4 | <0.0001 | **** |
| Type IIA | 19.05 | 3.7 | 16.9 | 3.6 | -11.3 | <0.0001 | **** | 28.56 | 6.6 | 25.54 | 6.3 | -10.6 | <0.0001 | **** |
| Type IIB | 34.06 | 6.8 | 31.8 | 6.3 | -6.6 | <0.0001 | **** | 31.29 | 7.7 | 22.64 | 6.1 | -27.6 | 0.0015 | ** |
| Type IIA/IIX | 19.72 | 3.9 | 17.97 | 3.7 | -8.9 | 0.006 | ** | 30.23 | 7.4 | 23.67 | 3.9 | -21.7 | <0.0001 | **** |
| **Proportions %** |  |  |  |  |  |  |  |  |  |  |  |  |  |  |
| Type I | 3.35 | 1.7 | 8.03 | 5.6 | +4.68 | 0.4 | ns | 35.26 | 5.8 | 48.63 | 4.9 | +13.37 | 0.1 | ns |
| Type IIA | 26.32 | 13.5 | 42.49 | 3.7 | +16.17 | 0.1 | ns | 48.52 | 7.9 | 46.85 | 4.8 | -1.67 | 0.7 | ns |
| Type IIB | 62.29 | 19.3 | 34.55 | 12.7 | -27.74 | 0.1 | ns | 5.78 | 2.3 | 0.86 | 1.5 | -4.92 | 0.1 | ns |
| Type I/IIA | 0 | 0 | 0 | 0 |  | - | - | 1.22 | 1.1 | 0 | 0 | -1.22 | 0.4 | ns |
| Type IIA/IIX | 8.05 | 4.4 | 14.92 | 9.4 | +6.87 | 0.4 | ns | 9.22 | 1.4 | 3.7 | 3.2 | -5.52 | 0.1 | ns |
