# Supplementary material for: Nebulin nemaline myopathy recapitulated in a compound heterozygous mouse model with both a missense and a nonsense mutation in Neb
Source: Acta Neuropathol Commun. 2020 Feb 17;8:18. doi: 10.1186/s40478-020-0893-1 (PMC7027239; doi:10.1186/s40478-020-0893-1)
Supplement: Supplementary file 2 — Additional file 2. Summary of the whole muscle physiology results. a Individual muscle mass, maximum specific force, twitch specific force, maximal rate of force production (dF/dt), contraction time (time-to-peak), half-relaxation time. Unpaired t-test. b Force frequency, c eccentric damage, d eccentric peak stretch. Two-way ANOVA (Sidak’s multiple comparisons test). Data presented as mean +/− SEM; n = 8; *p < 0.05; **p < 0.005; ***p < 0.0005; ****p < 0.0001. [file 40478_2020_893_MOESM2_ESM.docx]

**Supplementary Table 2. Summary of the whole muscle physiology results.**

**a** Individual muscle mass, maximum specific force, twitch specific force, maximal rate of force production (dF/dt), contraction time (time-to-peak), half-relaxation time. Unpaired t-test. **b** Force frequency, **c** eccentric damage, **d** eccentric peak stretch. Two-way ANOVA (Sidak's multiple comparisons test). Data presented as mean +/- SEM; n=8; *p < 0.05; **p < 0.005; ***p < 0.0005; ****p < 0.0001

**a**

|  | **EDL** | | | **SOL** | | |
| --- | --- | --- | --- | --- | --- | --- |
|  | **WT** | ***Neb*^Y2303H,Y935X^** | **p** | **WT** | ***Neb*^Y2303H,Y935X^** | **p** |
| Muscle mass (mg) | 10.7 ± 0.54 | 10.4 ± 0.2 | ns | 9.0 ± 0.40 | 9.4 ± 0.27 | ns |
| Maximum specific force (N/cm^2^) | 24.1 ± 0.47 | 23.3 ± 0.96 | ns | 22.9 ± 0.78 | 22.1 ± 0.87 | ns |
| Twitch specific force (N/cm^2^) | 6.1 ± 0.24 | 5.7 ± 0.24 | ns | 4.2 ± 0.18 | 3.9 ± 0.28 | ns |
| Max dF/dt (maximal rate of force production, g/s) | 1046.2 ± 54.39 | 1028.7 ± 58.96 | ns | 744.8 ± 41.21 | 928.9 ± 109.8 | ns |
| Time-to-peak (contraction time, ms) | 24.0 ± 0.53 | 21.3 ± 0.94 | 0.0234* | 42.0 ± 1.63 | 36.9 ± 1.87 | 0.0592 ns |
| Half-relaxation time (ms) | 26.6 ± 1.76 | 26.9 ± 0.81 | ns | 85.6 ± 5.22 | 71.86 ± 5.14 | ns |

**b Force-stimulation frequency relationship** (Fig. 5 a-c, normalised force)

| **EDL force-stimulation frequency, N/cm^2^** (*Neb*^Y2303H,Y935X^ – WT) | | | | | |
| --- | --- | --- | --- | --- | --- |
| Stimulation Frequency (Hz) | Mean difference | 95,00% CI of difference | Significant? | Summary | Adjusted P value |
| 10 | -0.9319 | -3.512 to 1.648 | No | ns | 0.9806 |
| 20 | -1.565 | -4.145 to 1.015 | No | ns | 0.6166 |
| 30 | -2.305 | -4.885 to 0.2744 | No | ns | 0.1167 |
| 40 | -1.726 | -4.306 to 0.854 | No | ns | 0.4727 |
| 60 | -1.123 | -3.703 to 1.457 | No | ns | 0.9283 |
| 70 | -1.063 | -3.643 to 1.517 | No | ns | 0.9501 |
| 80 | -1.042 | -3.622 to 1.538 | No | ns | 0.9565 |
| 100 | -0.9167 | -3.497 to 1.663 | No | ns | 0.9829 |
| 120 | -0.823 | -3.403 to 1.757 | No | ns | 0.9927 |
| 150 | -0.7711 | -3.351 to 1.809 | No | ns | 0.9958 |
| 200 | -0.7752 | -3.355 to 1.805 | No | ns | 0.9956 |

| **EDL force-stimulation frequency relationship, normalised as a relative force of max** (*Neb*^Y2303H.Y935X^ – WT) | | | | | |
| --- | --- | --- | --- | --- | --- |
| Stimulation Frequency (Hz) | Mean difference | 95.00% CI of difference | Significant? | Summary | Adjusted P value |
| 10 | -2.864 | -8.065 to 2.337 | No | ns | 0.7417 |
| 20 | -5.36 | -10.56 to -0.1587 | Yes | * | 0.0385 |
| 30 | -8.03 | -13.23 to -2.829 | Yes | *** | 0.0002 |
| 40 | -5.123 | -10.32 to 0.07755 | No | ns | 0.0566 |
| 60 | -2.077 | -7.278 to 3.124 | No | ns | 0.9597 |
| 70 | -1.674 | -6.875 to 3.527 | No | ns | 0.9922 |
| 80 | -1.423 | -6.624 to 3.778 | No | ns | 0.9981 |
| 100 | -0.6877 | -5.889 to 4.513 | No | ns | >0.9999 |
| 120 | -0.1603 | -5.361 to 5.041 | No | ns | >0.9999 |
| 150 | 0.06997 | -5.131 to 5.271 | No | ns | >0.9999 |
| 200 | -0.08573 | -5.287 to 5.115 | No | ns | >0.9999 |

| **SOL force-stimulation frequency, N/cm^2^** (*Neb*^Y2303H.Y935X^ – WT) | | | | | |
| --- | --- | --- | --- | --- | --- |
| Stimulation Frequency (Hz) | Mean difference | 95.00% CI of difference | Significant? | Summary | Adjusted P value |
| 5 | -0.8607 | -3.628 to 1.907 | No | ns | 0.9913 |
| 10 | -2.34 | -5.107 to 0.4279 | No | ns | 0.1618 |
| 15 | -2.827 | -5.595 to -0.05992 | Yes | * | 0.0418 |
| 20 | -2.746 | -5.514 to 0.02111 | No | ns | 0.0532 |
| 30 | -2.294 | -5.061 to 0.4736 | No | ns | 0.1810 |
| 40 | -1.986 | -4.754 to 0.7813 | No | ns | 0.3562 |
| 60 | -1.55 | -4.317 to 1.218 | No | ns | 0.6999 |
| 80 | -1.151 | -3.918 to 1.617 | No | ns | 0.9349 |
| 100 | -0.9014 | -3.669 to 1.866 | No | ns | 0.9877 |
| 120 | -0.7137 | -3.481 to 2.054 | No | ns | 0.9981 |

| **SOL force-stimulation frequency relationship, normalised as a relative force of max** (*Neb*^Y2303H.Y935X^ – WT) | | | | | |
| --- | --- | --- | --- | --- | --- |
| Stimulation Frequency (Hz) | Mean difference | 95.00% CI of difference | Significant? | Summary | Adjusted P value |
| 5 | -3.346 | -9.201 to 2.509 | No | ns | 0.6750 |
| 10 | -9.707 | -15.56 to -3.853 | Yes | **** | <0.0001 |
| 15 | -11.29 | -17.14 to -5.434 | Yes | **** | <0.0001 |
| 20 | -10.46 | -16.32 to -4.609 | Yes | **** | <0.0001 |
| 30 | -7.913 | -13.77 to -2.059 | Yes | ** | 0.0018 |
| 40 | -6.192 | -12.05 to -0.3372 | Yes | * | 0.0307 |
| 60 | -3.925 | -9.78 to 1.93 | No | ns | 0.4532 |
| 80 | -1.932 | -7.787 to 3.923 | No | ns | 0.9864 |
| 100 | -0.7039 | -6.559 to 5.151 | No | ns | >0.9999 |
| 120 | 0.1768 | -5.678 to 6.032 | No | ns | >0.9999 |

**c Eccentric damage** (Fig. 5 c-d)

| **EDL eccentric damage** (*Neb*^Y2303H.Y935X^ – WT) | | | | | |
| --- | --- | --- | --- | --- | --- |
| Stretch | Mean difference | 95.00% CI of difference | Significant? | Summary | Adjusted P value |
| 105% | -1.989 | -7.592 to 3.614 | No | ns | 0.9186 |
| 110% | -4.041 | -9.644 to 1.563 | No | ns | 0.2895 |
| 120% | -6.764 | -12.37 to -1.161 | Yes | ** | 0.0098 |
| 130% | -8.814 | -14.42 to -3.211 | Yes | *** | 0.0003 |
| 140 % | -8.034 | -13.64 to -2.43 | Yes | ** | 0.0013 |

| **SOL eccentric damage** (*Neb*^Y2303H.Y935X^ – WT) | | | | | |
| --- | --- | --- | --- | --- | --- |
| Stretch | Mean difference | 95.00% CI of difference | Significant? | Summary | Adjusted P value |
| 105% | -0.5815 | -5.913 to 4.75 | No | ns | 0.9998 |
| 110% | -1.337 | -6.668 to 3.995 | No | ns | 0.9845 |
| 120% | -0.7945 | -6.126 to 4.537 | No | ns | 0.9991 |
| 130% | 0.4924 | -4.839 to 5.824 | No | ns | >0.9999 |
| 140 % | 4.268 | -1.063 to 9.6 | No | ns | 0.1861 |

**d Eccentric-contraction peak force as a percentage of the previous isometric contraction** (Fig. 5 e-f)

| **EDL eccentric-contraction peak force** (*Neb*^Y2303H.Y935X^ – WT) | | | | | |
| --- | --- | --- | --- | --- | --- |
| Stretch | Mean difference | 95.00% CI of difference | Significant? | Summary | Adjusted P value |
| 5% | -3.703 | -16.75 to 9.344 | No | ns | 0.9524 |
| 10% | -3.195 | -16.24 to 9.852 | No | ns | 0.9745 |
| 20% | 2.357 | -10.69 to 15.4 | No | ns | 0.9935 |
| 30% | 13.01 | -0.0401 to 26.06 | No | ns | 0.0511 |
| 40 % | 19.64 | 6.595 to 32.69 | Yes | *** | 0.0008 |

| **SOL eccentric-contraction peak force** (*Neb*^Y2303H.Y935X^ – WT) | | | | | |
| --- | --- | --- | --- | --- | --- |
| Stretch | Mean difference | 95.00% CI of difference | Significant? | Summary | Adjusted P value |
| 5% | -11.24 | -35.86 to 13.38 | No | ns | 0.7318 |
| 10% | -15.37 | -39.99 to 9.253 | No | ns | 0.4207 |
| 20% | -15.24 | -39.86 to 9.383 | No | ns | 0.4300 |
| 30% | -20.02 | -44.64 to 4.604 | No | ns | 0.1640 |
| 40 % | -26.59 | -51.22 to -1.971 | Yes | * | 0.0283 |
