# Supplementary figures and images for: Nebulin nemaline myopathy recapitulated in a compound heterozygous mouse model with both a missense and a nonsense mutation in Neb
Source: Acta Neuropathol Commun. 2020 Feb 17;8:18. doi: 10.1186/s40478-020-0893-1 (PMC7027239; doi:10.1186/s40478-020-0893-1)

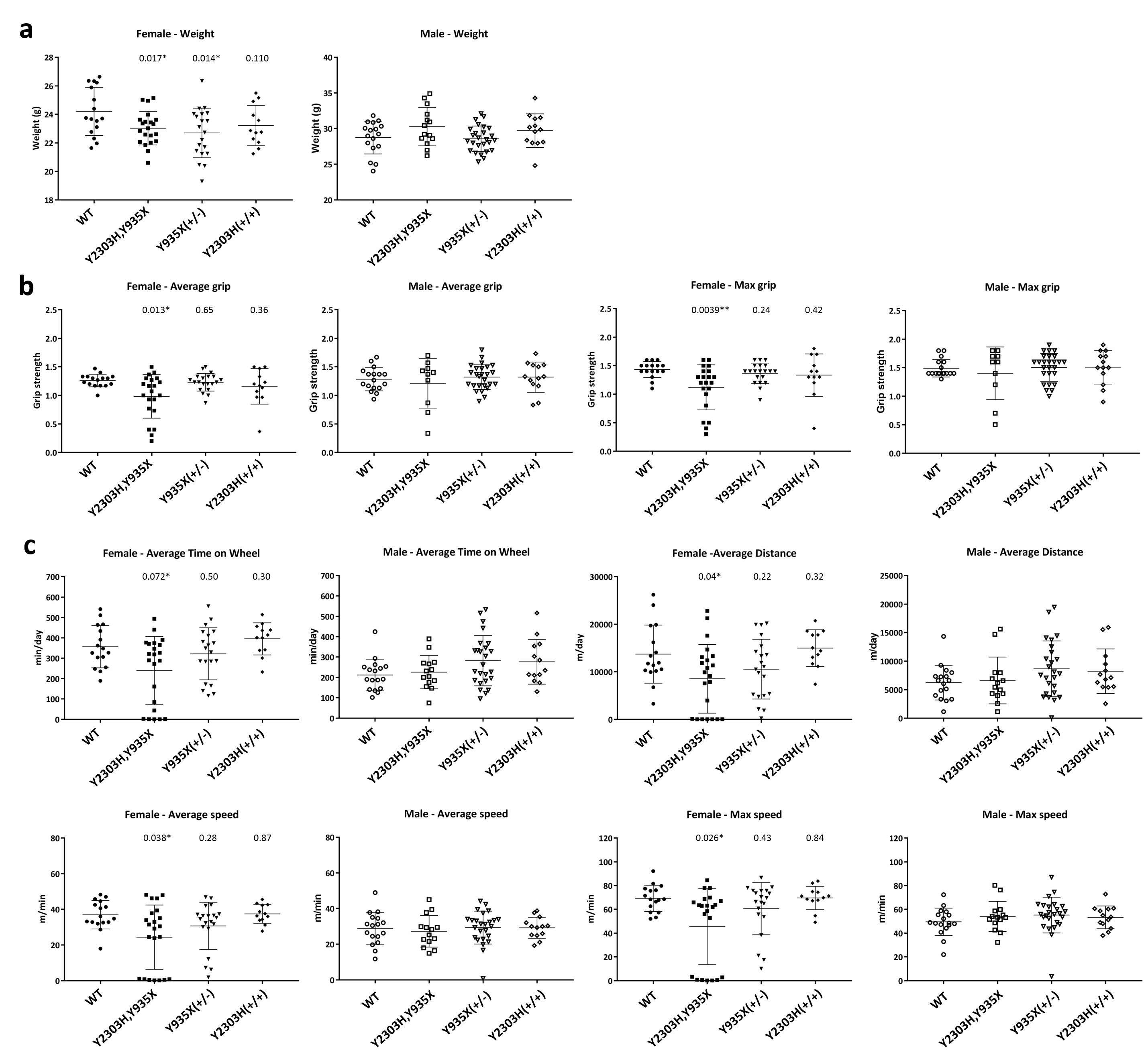

Supplement: Supplementary file 3 — Additional file 3. Bodyweight and gross functional changes of NebY2303H,Y935X, NebY935X and NebY2303H mice at 6 months of age. a Female NebY2303H,Y935X and NebY935X mice weighed less (p = 0.017* and p = 0.014*, respectively) than wild-type littermate controls. The functional performance of the mice was tested using whole animal in vivo experiments. b Grip strength testing showed reduced average grip (p = 0.013*) and reduced maximum grip (p = 0.0039**) for the female NebY2303H,Y935X mice. c Female NebY2303H,Y935X mice had impaired performance for three of the four voluntary running wheel measures; distance (p = 0.04*), max speed (p = 0.026*), duration (p = 0.072), and average speed (p = 0.038*). However, no significant differences were demonstrated with the rotarod analyses (data not shown), or for the males in any of the functional tests. Mann-Whitney, *p < 0.05; **p < 0.005, n = 10–22 for each parameter. [file 40478_2020_893_MOESM3_ESM.tif]
